# The polyunsaturated fatty acid and oxylipin plasma signature of aneurysmal subarachnoid haemorrhage, case-control study

**DOI:** 10.1016/j.neurot.2025.e00736

**Published:** 2025-09-10

**Authors:** M.A. Franssen, M.A. Tjerkstra, M. Heijink, S.A. Rotman, D. Verbaan, E. van Bavel, W.P. Vandertop, H.E. de Vries, J.M. Coutinho, M.A. Giera, I.A. Mulder, G. Kooij

**Affiliations:** a Department of Molecular Cell Biology and Immunology, Amsterdam UMC Location Vrije Universiteit Amsterdam, De Boelelaan 1117, Amsterdam, the Netherlands; b Amsterdam Neuroscience, Amsterdam UMC, Amsterdam, the Netherlands; c Department of Neurosurgery, Amsterdam UMC Location AMC, Amsterdam, the Netherlands; d Department of Biomedical Engineering & Physics, Amsterdam UMC Location AMC, Amsterdam, the Netherlands; e Department of Neurology, Amsterdam UMC Location AMC, Amsterdam, the Netherlands; f Centre for Proteomics and Metabolomics, Leiden UMC, Leiden, the Netherlands; g Amsterdam Cardiovascular Sciences, Amsterdam UMC, Amsterdam, the Netherlands; h Amsterdam Institute for Immunology and Infectious Diseases, Amsterdam UMC, Amsterdam, the Netherlands

**Keywords:** Lipidomics, Aneurysmal subarachnoid haemorrhage, Oxylipins, Polyunsaturated fatty acids, 12(S)-HETE, Plasma

## Abstract

Delayed cerebral ischemia (DCI) following aneurysmal subarachnoid haemorrhage (aSAH) is a complex and acute condition with limited options for early detection and effective treatments. The plasma levels of individual polyunsaturated fatty acids (PUFA) and their bioactive metabolites (oxylipins) of aSAH patients both at admission and over time remain largely unexplored, particularly concerning the development of DCI. In this study, plasma samples of aSAH patients were collected at admission and on days 4, 10, and 21 post-admission. ASAH patients who did not develop DCI were age- and sex matched to aSAH patients who did develop DCI. Control groups included patients with an unruptured aneurysm (UA) and healthy controls (HC). PUFA and oxylipin levels in plasma were measured using liquid chromatography with tandem mass spectrometry and were analysed using non-parametric univariate tests. At admission, aSAH (n ​= ​47) patients showed elevated levels of several PUFAs, such as linoleic acid and arachidonic acid, as well as oxylipins, including 12-HETE, 20-HETE and 19,20-DiHDPA, compared to UA (n ​= ​24) and HC (n ​= ​13). 12-HETE was predominantly found in the *S*-configuration, indicating synthesis via 12(*S*)-lipoxygenase. PUFA and oxylipin levels dropped significantly by day four post-admission, except for 19,20-DiHDPA. No PUFAs or oxylipins differentiated patients who developed DCI. We characterized a distinct plasma PUFA- and oxylipin profile in aSAH patients at admission and identified a significant decline in PUFA and oxylipin levels by day 4 post-admission.

## Introduction

Aneurysmal subarachnoid haemorrhage (aSAH) is a severe subtype of stroke, which is caused by the rupture of an intracranial aneurysm, leading to high disability rates and case fatality [1,2]. Early brain injury (EBI) occurs within the first 72 ​h, driven by intracranial pressure changes, neuroinflammation and vascular dysfunction [3]. Delayed cerebral ischemia (DCI), occurring in approximately 30% of patients 4–14 days post-haemorrhage, further worsens outcomes [4,5]. Both conditions involve multifactorial mechanisms, yet reliable biomarkers for early diagnosis and treatment guidance remain elusive [6,7].

Polyunsaturated fatty acids (PUFAs) and their bioactive lipid metabolites (oxylipins) play crucial roles in inflammation and vascular tone regulation, making them promising biomarker candidates [8,9]. PUFAs are released from phospholipids by the phospholipase A2 (PLA2) enzyme family [10] and subsequently metabolized into oxylipins by cyclooxygenase (COX), lipoxygenase (LOX) and cytochrome P450 (CYP) enzymes to their respective oxylipins [11]. With the advances in the metabolomic field and techniques like liquid chromatography-tandem mass spectrometry (LC-MS/MS), various PUFAs and oxylipins levels can now be reliably studied [12]. Accordingly, increased levels of omega-3 and omega-6 (*n*-3 and *n*-6, respectively) free fatty acids (FFA) were observed in the serum and plasma of aSAH patients [13,14]. Similarly, studies have identified PUFAs and oxylipins in the cerebrospinal fluid (CSF) of aSAH patients [[15], [16], [17], [18]]. However, the detailed plasma profile of PUFAs and oxylipins, especially longitudinally and in relation to DCI, remains underexplored. Previous evidence in rodent models suggests that specific AA-derived oxylipins are associated with vascular reactivity [19], while DHA-derived oxylipins have shown beneficial potential in reducing oedema and limiting BBB permeability [20]. Supplementation with DHA and eicosapentaenoic acid (EPA) in aSAH patients after surgical clip application or coil embolization has also been associated with reduced occurrence of vasospasm and improved functional outcomes [21].

This study aims to investigate plasma PUFA and oxylipin profiles in aSAH patients compared to healthy controls (HC), as well as patients with an unruptured aneurysm (UA). Plasma samples were collected at admission and at 4-, 10- and 21 days post-admission, and measured using targeted LC-MS/MS. We hypothesized that PUFA and oxylipin levels at admission would differentiate aSAH patients from controls and that their longitudinal changes would correlate with DCI development, disease severity and clinical outcomes.

## Methods

### Study population

Blood plasma samples of aSAH patients were acquired in collaboration with an ongoing, prospective, observational study on platelet RNA profiles in aSAH patients conducted at the Amsterdam University Medical Centre (UMC), the Netherlands. The samples were collected from June 2018 until February 2021. Detailed in- and exclusion criteria can be found in Supplementary Table 1. The standard treatment focussed on securing the aneurysm within 24–72 ​h, using endovascular coiling when feasible or surgical clipping as an alternative. All patients received 2850 IU of fraxiparin as a preventive measure against thrombosis. Patients that developed clinical DCI within 2 weeks of admission were included in the aSAH ​+ ​DCI group. These aSAH ​+ ​DCI patients were then age- and sex-matched with patients who did not develop clinical DCI (aSAH-DCI). Blood plasma of UA patients was collected from patients when they visited the outpatient clinic or patients admitted to the hospital for treatment.

### Standard protocol approvals, registrations, patient consents and data availability statement

Written informed consent or deferred consent was obtained of all aSAH and UA patients, as well as HC. The study was conducted in accordance with the Declaration of Helsinki and was approved by the medical-ethical committee of the Amsterdam UMC (location Meibergdreef, approval no. 2017_318). Any data not provided in the article may be shared (anonymised) at the request of any qualified investigator for purposes of replicating procedures and results.

### Demographic and clinical data

Prospectively collected data from the institution's SAH registry was utilized, including age at diagnosis or first blood withdrawal, sex, body mass index (BMI), smoking status, pre-admission medication use and comorbidities (hypertension, hypercholesterolemia, diabetes mellitus). We additionally collected the neurological status of aSAH patients at admission, as graded by the World Federation of Neurological Surgeons (WFNS) scale, amount and distribution of subarachnoid blood on CT as graded by the Fisher scale, location and treatment of the ruptured aneurysm, the occurrence of DCI, in-hospital case fatality, 6-months’ case fatality and the functional outcome at six months after aSAH, as assessed by the modified Rankin Scale (mRS) score. Clinical and radiological DCI were defined according to Vergouwen et al. [22]. In short, the definition of clinical DCI was a decline in consciousness or focal neurological deficits lasting at least 1 ​h, with no other identifiable cause. Radiological DCI was defined as a new cerebral infarction on CT- or MR-imaging, that cannot be rooted in treatment of the aneurysm or other causes and was not present on CT- or MR-imaging 24–48 ​h after SAH.

### Sample collection

Peripheral blood of aSAH patients was collected upon admission (day 0), on day 4 [[3], [4], [5]] and on day 10 [[9], [10], [11]] and day 21 [[20], [21], [22]] in EDTA tubes and was spun down within 12 ​h after collection at 120G for 20 ​min. After that, the platelet-rich fraction was isolated and centrifuged at 360G for 20 ​min. Then, platelet-poor plasma was isolated and centrifuged at 3300G for 10 ​min, after which plasma was isolated and stored at −80 ​°C until further use. Of both control groups, one sample was collected and centrifuged similarly.

### Targeted lipidomic sample processing

Before processing the plasma samples, they were first randomized by a researcher not involved in any steps of the measurements or analysis. The researcher performing the measurements and preprocessing was blinded. Sample preparation was performed as previously described [23], with minor adaptions. To generate quality control samples, 50 ​μL from each biological sample was pooled and aliquoted into 24 samples of 350 ​μL each. Quality controls were included in every tenth sample during solid-phase extraction (SPE) and LC-MS/MS auto-sampling. In short, protein precipitation was done by adding 1350 ​μL of ice-cold methanol (Merck, Darmstadt, Germany) containing 4 ​μL internal standard solution to 350 ​μL of plasma (deuterated oxylipins: leukotriene B_4_-d4 (50 ​ng/mL), 15-hydroxyeicosatetraenoic acid-d8 (50 ​ng/mL), prostaglandin E_2_-d4 (50 ​ng/mL), 8-iso prostaglandin F2α-d4 (100 ​ng/mL), docosahexaenoic acid-d5 (500 ​ng/mL), and 14(15)-epoxy-eicosatrienoic acid-d11 (50 ​ng/mL) (Cayman Chemical Ann Arbour, MI, USA)). Thereafter, samples were centrifuged at 16,100 ​× ​G and 4 ​°C for 10 ​min. Supernatants were transferred to 15 ​mL tubes, diluted with 7.5 ​mL water, and acidified to pH 3.5 with 10 ​% formic acid (84865.180, VWR). SPE with C-18 cartridges (Sep-Pak, Vac3 3 ​cc (200 ​mg), Waters, MA, USA)) was used to extract lipids. Samples were cleaned with LC-MS grade water (Biosolve BV, Valkenswaard, the Netherlands) and n-hexane (Honeywell-Riedel de Haeenee4ae, Seelze, Germany), eluted with methylformate (259705, Honeywell-Riedel de Haën, Seelze, Germany), and dried at 40 ​°C under nitrogen. Dried samples were reconstituted in 80 ​μL LC-MS grade methanol (Merck, Darmstad, Germany) and 120 ​μL ​LC-MS grade water (Honeywell-Riedel de Haen, Seelze, Germany), then transferred to deactivated glass inserts (Agilent, CA). Finally, 40 ​μL of the solution was injected into the High Performance Liquid Chromatography (HPLC)-system (Shimadzu, The Netherlands). The HPLC system used a C18 column with a C8 precolumn. Column temperature was maintained at 50 ​°C. Lipids were separated by a binary gradient of water (A) and methanol (B) containing 0.01 ​% acetic acid. The gradient was generated as follows: 0 ​min 30 ​% B, held for 1 ​min, then ramped to 45 ​% B at 1.1 ​min, 53.5 ​% B at2 min, 55.5 ​% B at 4 ​min, 90 ​% B at 7 ​min, and 100 ​% B at 7.1 ​min, and held for 1.9 ​min. The injection volume was 40 ​μl, and the flow rate was 400 ​μL/min. A Sciex Qtrap 6500 tandem mass spectrometer (MS/MS) in negative electrospray ionization mode was used to detect individual PUFA's and oxylipins (Multiple Reaction Monitoring settings can be found in Supplementary Table 2). The needle voltage was set to −4500 ​V, the drying temperature to 450 ​°C, ion source gas 1/nebulizer gas (air) to 40 psi, the ion source gas 2/drying gas (air) to 30 psi, and the nebulizer gas (nitrogen) to 30 psi. Peak identification was performed using SciexOS® Software. The relative abundance was defined relative to the internal standard (area ratio) and the absolute concentrations were determined for 27of the 77 lipid species using external calibration curves. The calculated concentrations of all groups and at every measured timepoint of those PUFAs and oxylipins for which a calibration curve was available are presented in Supplementary Table 3.

### Chiral analysis

For chirality measurements, we obtained an additional 200 ​μL EDTA-plasma aliquots taken at admission of six aSAH patients that were measured with LC-MS/MS to be compared with UA and HC in this study, based on the availably of the respective aliquots. To 200 ​μL of plasma aliquots, 600 ​μL methanol and 4 ​μL of a 100 ​ng/mL internal standard (15*S*-HETE-d8 (100 ​μg/mL in acetonitrile (ACN)), (±) 5-HETE (100ug/mL in MeOH), (±) 11-HETE (100ug/mL in MeOH), (±) 12-HETE (100ug/mL in MeOH), (±) 15-HETE (100 ​μg/mL in MeOH) (Cayman Chemical Ann Arbour, MI, USA)) was added and vortexed. After incubation at −20 ​°C for 20 ​min, protein precipitation was done by centrifugation at 16,100 x G for 10 ​min. The supernatant was added to 2 ​mL HPLC grade water with 0.5 ​μl of formic acid. Samples were then subjected to SPE (C-18 cartridges (Sep-Pak, Vac3 3 ​cc (200 ​mg), Waters)). Samples were washed with water and n-hexane (Honeywell-Riedel de Haen, Seelze, Germany), eluted with methylformate (259705, Honeywell-Riedel de Haën, Seelze, Germany), and dried at 40 ​°C under nitrogen. Dried samples were reconstituted in 100 ​μL of a methanol and water mixture (60:40, v/v%), vortexed and sonicated, then transferred to deactivated glass inserts. Until further measurement, the sample was stored at −80 ​°C.

Further purification by fractionation was done using a SCIEX Qtrap 6500+ LC-MS/MS instrument. The HPLC was performed using a reversed phase XSelect® CSHTM C18 column (2.1 ​× ​100 ​mm, 3.5 ​μm, Waters, Ireland) with a constant flowrate of 500 ​μL/min, and mobile phases consisting of solvent A (water with 0.01 ​% acetic acid) and B (methanol with 0.01 ​% acetic acid). For isolation and collection of HETEs the method was set to isocratic mode at 90%B, with a column temperature of 50 ​°C and an injection volume of 50 ​μL. Initially, MS detection was performed in negative mode using MRM of selected Q1 and Q3 masses to identify retention times for fractionation (Supplementary Table 4). Fractions corresponding to HETEs were collected between 0.5 and 2.0 ​min. Collected fractions were dried under nitrogen and stored in a freezer until further analysis. For chiral analysis, in the collected fraction was reconstituted in 100 ​μL of a LC-MS grade methanol/water mixture (60:40, v/v%). Analysis was executed on a HPLC-MS/MS (LC-40 HPLC system (Shimadzu, The Netherlands), QTRAP 6500+ MS System (SCIEX, The Netherlands) using a CHIRALPAK® AD-RH chiral column (2.1 ​× ​150 ​mm, 5 ​μm, Daicel, Japan), with a total flowrate of 300 ​μL/min, and gradient of 50 ​% B to 85 ​% B over 6 ​min, held over 4 ​min with mobile phases consisting of A: water with 0.01 ​% acetic acid, and B: ACN with 0.01 ​% acetic acid. The column temperature was set at 50 ​°C and the injection volume was 20 ​μL.

### Pre-processing of LC-MS/MS data

Components were considered reliably detected and considered for further analysis if present in at least 80 ​% of all samples within one group, according to the adjusted 80 ​% ruling [24]. The remaining missing values were imputed per group and timepoint using a Gibbs sampler based left-censored imputation method for targeted lipidomic analysis, as described previously [25].

### Statistical analyses

Baseline characteristics were reported as means ​± ​standard deviations (SD), medians with interquartile ranges (Q1-Q3), or relative proportions. Normality of continuous variables was assessed visually and via the Shapiro-Wilk test. Outliers were identified using median absolute deviations but were not excluded for analysis with exception of the ordinal regression models. Statistical analysis and graphing were performed in R-Studio (Version 2024.09.0 ​+ ​375) using ggplot2, rStatix, MASS, polr, and Hmisc. For age and years of smoking, ANOVA and independent t-tests were used. Other baseline characteristics were analysed with Kruskal-Wallis or Mann-Whitney U tests. A potential association sex with PUFA and oxylipin levels was investigated by applying a Mann-Whitney U test for aSAH patients at every timepoint and the combined control groups. PUFA and oxylipin levels in aSAH patients were compared to UA and HC groups via Mann-Whitney U tests (H1: aSAH ≠ UA; aSAH ≠ HC), with fold changes derived from median log2 foldchanges. Longitudinal PUFA/oxylipin changes were assessed using paired Wilcoxon signed-rank tests at each timepoint (H1: T0 ≠ T1; T1 ≠ T2; T2 ≠ T3). Significance testing used non-normalised levels. Ordinal regression was used to assess relationships between PUFA/oxylipin levels and mRS at six months, the modified Fisher score, the NIHSS at each respective timepoints (H1: there is a significant relationship between the predictor variables and the odds of being in a particular category or lower). To investigate the association of PUFA/oxylipin levels with plasma neurofilament light chain (NfL) as well as age, a robust linear model was fitted. Plasma NfL were log transformed before the linear regression. Coefficients are described before and after backtransformation (exp(ß)). Model diagnostics included visual inspection of Q-Q plots to evaluate normality of residuals as well as residuals vs fitted values to assess linearity and homoscedasticity. Residuals vs leverage plots with Cook's distance contours to detect influential observation. Kruskal-Wallis tests examined associations with treatment modality, aneurysm location, and WFNS grade (H1: unequal mean ranks). The relationship with the modified Fisher score was analysed using ordinal regression. Differences in PUFA/oxylipin levels between DCI and non-DCI patients were tested with Mann-Whitney U tests (H1: aSAH ​+ ​DCI ≠ aSAH-DCI). All p-values, except those generated were adjusted for false discovery rate by means of Benjamini & Hochberg (BH) method [26] or Benjamini-Yekutieli (BY) procedure [27]*.* The significance level was set at α ​= ​0.05. Both adjusted and non-adjusted p-values are reported.

## Results

### Patient characteristics

**Fig. 1:**
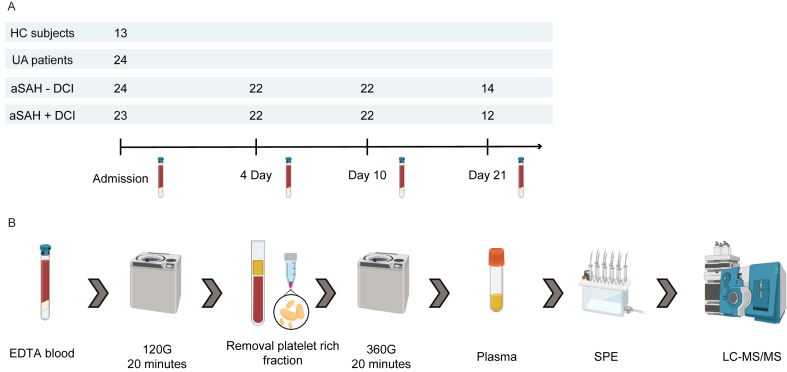
Infographic of the study design. A) Overview of the number of samples available for analysis per timepoint of plasma collection for aneurysmal subarachnoid haemorrhage (aSAH) patients with (+DCI) and without delayed cerebral ischemia (-DCI), patients with an unruptured aneurysm (UA) and healthy controls (HC). B) Experimental methods showing the processing of the EDTA plasma samples, more specially the centrifugation steps, solid phase extraction (SPE) and measurement using liquid chromatography-tandem mass spectrometry (LC-MS/MS).

A total of 23 aSAH ​+ ​DCI patients were enrolled in this study, which were matched to 24 aSAH-DCI patients. At the timepoints day 4 and 10, two samples in the aSAH ​+ ​DCI group and one sample in the aSAH-DCI group had to be excluded due to haemolysis or insufficient signal during LC-MS/MS measurement (Fig. 1A and B). For the last timepoint at day 21, 12 samples were available for the aSAH ​+ ​DCI patients and 14 for the aSAH-DCI patients (Fig. 1A). As for the controls, EDTA plasma samples of 13 HC and 24 UA patients were measured using LC-MS/MS (Fig. 1A and B). In this cohort, one-way ANOVA comparing age across the different patient groups showed no difference at baseline (F ​= ​2.497, p ​= ​0.066), and a Tukey HSD test revealed no significant differences in age between any of the groups in this study (Supplementary Fig. 1A). UA patients had a mean age of 60.5 years (SD 10.2) compared to aSAH ​+ ​DCI (51.6 (±15.1)), aSAH-DCI (51.7 (±11.6)) and healthy controls (53.00 ​± ​8.2) (Table 1). aSAH patients were 87 ​% female with a median BMI of 26 (IQR 7.2), while UA patients were 70 ​% female with a median BMI of 25.4 (IQR 5.0) and HC were 92 ​% female (Table 1). The median duration of hospitalization was 22 days for aSAH ​+ ​DCI patients (IQR 8.5) and 21 (IQR 15.0) days for aSAH-DCI patients (IQR 15.0), with 4 patients dying in the aSAH ​+ ​DCI group and none in the aSAH-DCI group (Supplementary Fig. 1B). Four (18 ​%) aSAH patients developed DCI before day five and 20 (90 ​%) of the aSAH patients developed DCI before 11 days of hospitalization (Supplementary Fig. 1C). While the NIHSS showed a median of 1 for both the aSAH ​+ ​DCI (IQR 11.75) and aSAH-DCI group (IQR 12.5), five aSAH ​+ ​DCI patients and three aSAH-DCI patients presented a NIHSS higher than 16 (Supplementary Fig. 1D). Sixty percent of aSAH patients presented a WFNS score of 1–2, while 98 ​% of all aSAH patients were assessed to have a modified Fisher score of 3–4 (Table 1). Of the 23 aSAH patients with clinical DCI, seven patients had radiological DCI. In the aSAH-DCI group, no patients had radiological DCI. Neither incidence of common complications, aneurysm location, functional outcome at 6-month follow-up, treatment of the ruptured aneurysm, nor pre-admission medication usage (except for protein pump inhibitor usage; p ​= ​0.003) differed significantly between aSAH ​+ ​DCI and aSAH-DCI patients (Table 1). For one aSAH ​+ ​DCI patient the functional outcome at 6 months follow-up was not available.

**Table 1:** Baseline table with demographics of aneurysmal subarachnoid haemorrhage (aSAH) patients with delayed cerebral ischemia (+DCI), without DCI (-DCI), patients with an unruptured aneurysm (UA) and healthy controls (HC). BMI = body mass index, mRS ​= ​modified Ranking Scale at 6 months, NIHSS = NIH stroke scale, WFNS = World Federation of Neurosurgical Societies, SD ​= ​standard deviation, IQR ​= ​interquartile range. All tests are either Mann–Whitney U test/l Kruskal–Wallis test except for those denoted with †, which denotes an ANOVA test.

	SAH	+ DCI	- DCI	UA	C	P
Mean age (±SD)	52.5 (±13.7)	51.7 (±15.5)	53.4 (±12.3)	60.5 (±10.2)	53.00 (±8.2)	0.043 †
n female (%)	41 (87 ​%)	20 (87 ​%)	21 (88 ​%)	17 (70 ​%)	12 (92 ​%)	0.22 ∗
BMI (IQR)	26 (7.2)	26.0 (5.6)	25.8 (8.4)	25.4 (4.3)	–	0.78 ∗
Diabetes mellitus type 2 n (%)	0 ​%	0 ​%	0 ​%	3 (4 ​%)	–	0.34 ∗
Hypertension n (%)	11 (23 ​%)	5 (21 ​%)	6 (25 ​%)	11 (45 ​%)	–	0.081∗
Smoking n (%)	37 (79 ​%)	22 (95 ​%)	15 (62 ​%)	21 (87 ​%)	–	0.6 ∗
Mean years of smoking (±SD)	21.5 (±14.8)	18.3 (±15.1)	23.5 (±15.1)	26.7 (±16.7)	–	0.76 †
Days of hospitalization (IQR)	22 days (12.5)	22 days (8.5)	21 days (15.0)	–	–	0.55 ∗
Mean NIHSS at admission (SD)	7.4 (10.8)	7.7 (12.2)	7 (9.3)	–	–	–
Median NIHSS at admission (IQR)	1 (12.5)	1 (6.0)	1 (12.5)	–	–	0.8 ∗
Radiological DCI	7	7	0	–	–	–
**Medication use**	**SAH**	**+ DCI**	**- DCI**			
Pain mediaction (not NSAID)	1	0	1	–	–	0.31∗
NSAID	5	3	2	–	–	0.63 ∗
Psychotropics	5	3	2	–	–	0.63 ∗
Corticosteroids	1	0	1	–	–	0.31 ∗
Antihypertensives	6	4	2	–	–	0.38 ∗
Proton pump inhibitors	4	4	0	–	–	0.03 ∗
**Complications**	**SAH**	**+ DCI**	**- DCI**			
Meningitis (%)	15 (31 ​%)	8 (34 ​%)	7 (30 ​%)	–	–	0.94 ∗
Urinary tract infection (%)	5 (10 ​%)	2 (9 ​%)	3 (13 ​%)	–	–	0.96 ∗
Pneumonia (%)	8 (17 ​%)	4 (18 ​%)	4 (17 ​%)	–	–	0.67 ∗
Sepsis (%)	0	0	0	–	–	–
Delirium (%)	0	0	0	–	–	–
**WFNS score at admission**	**SAH**	**+ DCI**	**- DCI**			0.52 ∗
1	28	10	9	–	–	
2	11	5	6	–	–	
3	3	2	1	–	–	
4	5	–	5	–	–	
5	9	6	3	–	–	
**Modified Fisher scale at admission**	**SAH**	**+ DCI**	**- DCI**			0.52 ∗
1	1	–	1	–	–	
2	0	–	–	–	–	
3	11	5	6	–	–	
4	35	18	17	–	–	
**Location of the ruptured aneurysm**	**SAH**	**+ DCI**	**- DCI**			0.79 ∗
A. Communicans anterior	16	7	9	–	–	
A. Communicans posterior	6	4	2	–	–	
A. Cerebri media	12	6	6	–	–	
Carotis interna	4	4		–	–	
A. Basilaris	3	1	2	–	–	
Carotis interna	5	–	5	–	–	
A. Vertebralis	1	1	–	–	–	
**Treatment of the ruptured aneurysm**	**SAH**	**+ DCI**	**- DCI**			0.74 ∗
Coiling	36	18	18	–	–	
Clipping	5	2	3	–	–	
Flow diverter	2	2	–	–	–	
Web device	1	–	1	–	–	
Parent vessel occlusion	1	–	1	–	–	
**MRS at 6 months follow up**	**SAH**	**+ DCI**	**- DCI**			0.66 ∗
0	1	–	1	–	–	
1	4	1	3	–	–	
2	22	11	11	–	–	
3	5	3	2	–	–	
4	5	3	2	–	–	
5	5	–	5	–	–	
Death	4	4	–	–	–	
Missing	1	1	–	–	–	

### No association of plasma PUFA and oxylipin levels with sex or age

To understand whether there are sex-associated differences in plasma levels of PUFAs or oxylipins contrasting men and women in aSAH patients and controls, Mann-Whitney U tests with BH correction were performed. Considering the small number of men in the HC (n ​= ​1) and UA patients (n ​= ​6) we pooled these 2 control groups to allow for sensible hypothesis testing. No differences were found between men and women in PUFAs and oxylipins levels of aSAH patients at admission, aSAH patients at any following timepoint nor the pooled control group (Supplementary Fig. 2A and B). Similarly, a robust linear regression with sex as covariate did not reveal any association of age with plasma PUFAs and oxylipin at any timepoint in aSAH patients (Supplementary Table 5).

### ASAH patients at admission display increased levels of PUFAs and oxylipins within both the *n*-6- and *n*-3-fatty acid metabolism compared to UA and HC

**Fig. 2:**
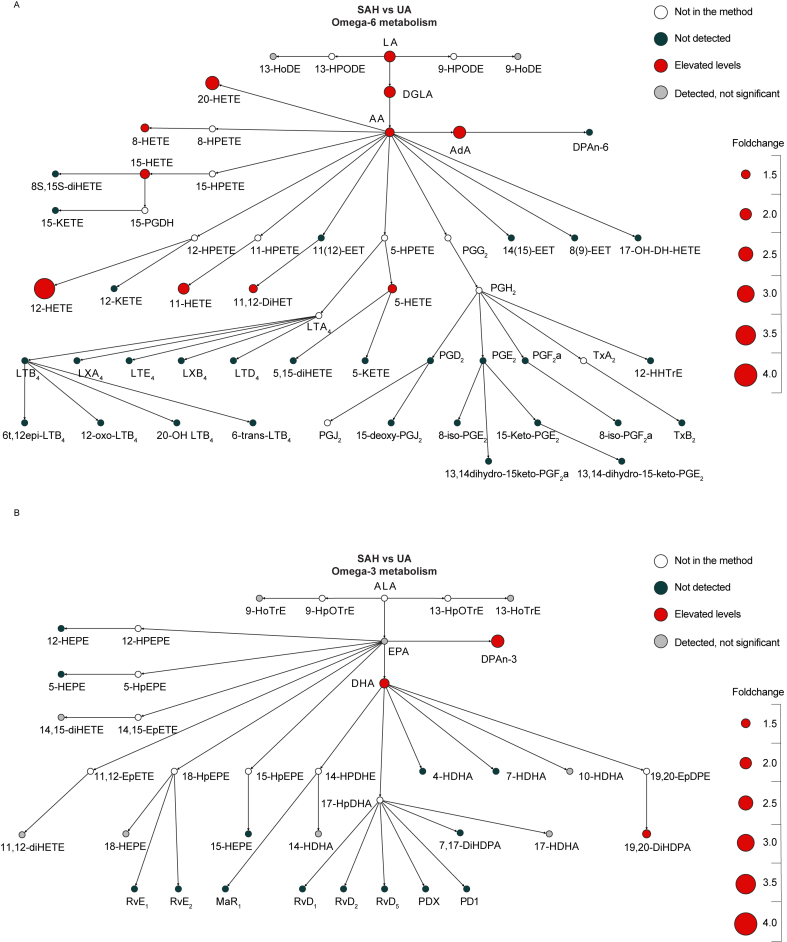
Changes in *n*3-and *n*6-polyunsatured fatty acids and oxylipins in aneurysmal subarachnoid haemorrhage (aSAH) patients at admission compared to patients with an unruptured aneurysm (UA). Colour indicates whether the component was in the method, not detected or differentially expressed (Mann–Whitney U test, corrected for multiple testing with Benjamini-Hochberg procedure). Size corresponds to the foldchange based on medians. A) Comparison of *n*6-PUFAs and -oxylipins levels in aSAH patients and UA. B) Comparison of *n*3-PUFAs and -oxylipins levels in aSAH patients and UA.

To investigate whether aSAH patients have altered PUFA- and oxylipin profiles, plasma samples of aSAH at admission, HC and UA were analysed using LC-MS/MS. We observed higher levels of *n*-6-PUFAs LA (median fold change (FC) ​= ​1.88; W ​= ​882; p adj. ​< ​0.001), AA (median fold change ​= ​1.50; W ​= ​751; p adj. ​= ​0.039), DGLA (median fold change (FC) ​= ​1,93; W ​= ​837; p adj. 0.003), AA (median fold change ​= ​1.50; W ​= ​751; p adj. ​= ​0.038), AA (median fold change ​= ​1.50; W ​= ​751; p adj. ​= ​0.038) and AdA (median FC ​= ​2.14; W ​= ​848; p adj. ​= ​0.001) when comparing aSAH at admission to UA (Fig. 2A and Supplementary Fig. 3A–X). Similarly, oxylipins originating from AA like 11,12-DiHET (median FC ​= ​1.90; W ​= ​889; p adj. ​< ​0.001), 5-HETE (median FC ​= ​1.44; W ​= ​771; p adj. ​= ​0.027), 8-HETE (median FC ​= ​1.44; W ​= ​771; p adj. ​= ​0.027), 11-HETE (median FC ​= ​1.39; W ​= ​765; p adj. ​= ​0.030), 12-HETE (median FC ​= ​3.64; W ​= ​955; p adj. ​< ​0.001), 15-HETE (median FC ​= ​1.60; W ​= ​831; p adj. ​= ​0.003) and 20-HETE (median FC ​= ​2.35; W ​= ​950; p adj. ​< ​0.001) displayed elevated levels in aSAH patients when compared to UA patients. Within the *n*-3-pathway, DHA (median FC ​= ​1.61; W ​= ​771; p adj. ​= ​0.027), DPAn-3 (median FC ​= ​1.61; W ​= ​771; p adj. ​= ​0.027) and the oxylipin 19,20-DiHDPA (median FC ​= ​2.18; W ​= ​837; p adj. ​= ​0.002) displayed higher levels in aSAH at admission compared to UA (Fig. 2B and Supplementary Fig. 3A–X).

**Fig. 3:**
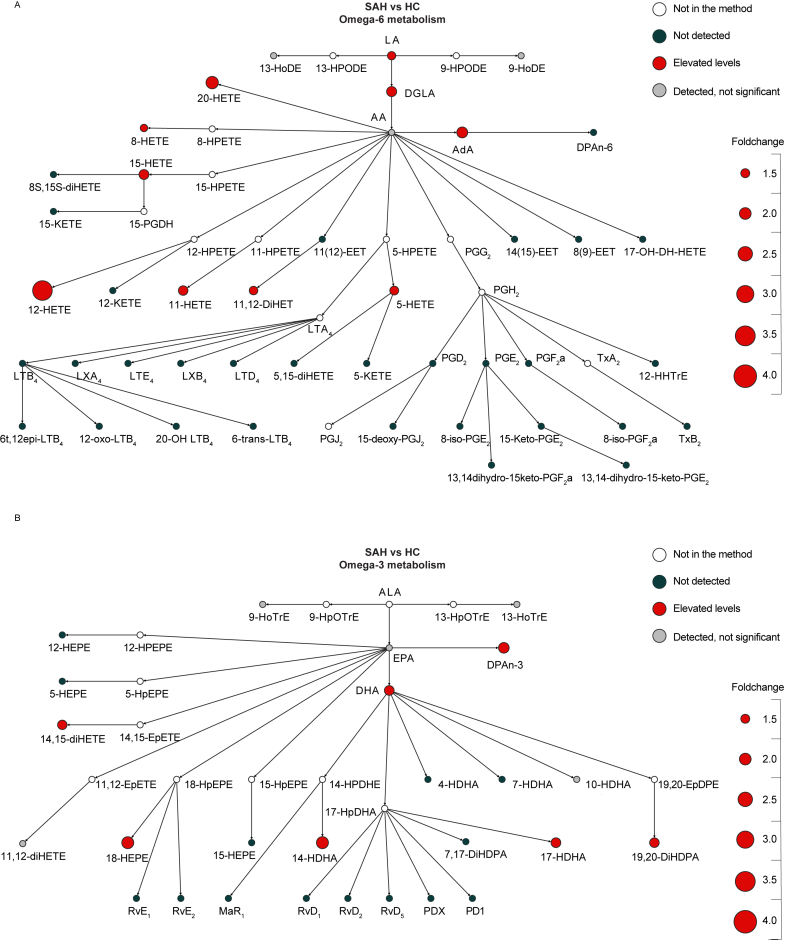
Changes in *n*3-and *n*6-polyunsatured fatty acids and oxylipins in aneurysmal subarachnoid haemorrhage (aSAH) patients at admission compared to healthy controls (HC). Colour indicates whether the component was in the method, not detected or differentially expressed (Mann–Whitney U test corrected for multiple testing with Benjamini-Hochberg procedure). Size corresponds to the foldchange based on medians. A) Comparison of *n*6-PUFAs and -oxylipins levels in aSAH patients and HC. B) Comparison of *n*3-PUFAs and -oxylipins levels in aSAH patients and HC.

Compared to HC, aSAH patients at admission revealed induced levels of LA (median FC ​= ​1.38; W ​= ​130; p adj. ​= ​0.005), DGLA (median FC ​= ​1.70; W ​= ​164; p adj. ​= ​0.024) and AdA (median FC ​= ​1.85; W ​= ​150; p adj. ​= ​0.017) in the *n*-6-pathway (Fig. 3A and Supplementary Fig. 3A–X). Moreover, the *n*-6-derived oxylipins 11,12-DiHET (median FC ​= ​1.60; W ​= ​101; p adj. ​< ​0.001), 5-HETE (median FC ​= ​1.48; W ​= ​163; p adj. ​= ​0.024), 8-HETE (median FC ​= ​1.23; W ​= ​172; p adj. ​= ​0.031), 11-HETE (median FC ​= ​1.67; W ​= ​94; p adj. ​= ​0.040), 12-HETE (median FC ​= ​3.45; W ​= ​60; p adj. ​< ​0.001), 15-HETE (median FC ​= ​3.45; W ​= ​60; p adj. ​< ​0.001), 20-HETE (median FC ​= ​2.13; W ​= ​121; p adj. ​= ​0.003) displayed higher levels in aSAH compared to HC. Within the *n*-3-metabolism, we observed higher levels of the PUFAs DHA (median FC ​= ​2.13; W ​= ​121; p adj. ​= ​0.003) and DPA-*n*-3 (median FC ​= ​1.81; W ​= ​161; p adj. ​= ​0.024) in aSAH patients compared to HC, as well as the *n*-3-derived oxylipins 14-HDHA (median FC ​= ​1.58; W ​= ​182; p adj. ​= ​0.041), 17-HDHA (median FC ​= ​1.65; W ​= ​174; p adj. ​= ​0.032), 19,20-DiHDPA (median FC ​= ​1.61; W ​= ​186; p adj. ​= ​0.047), 14,15-diHETE (median FC ​= ​2.05; W ​= ​166; p adj. ​= ​0.024) and 18-HEPE (median FC ​= ​2.05; W ​= ​162; p adj. ​= ​0.024) (Fig. 3B and Supplementary Fig. 3A–X). The medians, IQR and test statistics of the respective comparisons of all PUFAs and oxylipins can be found in Supplementary Table 6.

Within the *n*-6 metabolism of aSAH, 12-HETE exhibited the most prominent increase in foldchange of 3.6 and 3.4 compared to both UA and HC respectively. In the *n*-3-pathway, DPA-*n*-3 showed the largest increase in foldchange compared to UA, while 14-HDHA and 18-HEPE showed the highest foldchange increase when comparing aSAH to HC. Levels of neither prostaglandins nor leukotrienes were detected in the plasma of aSAH patients nor either of the control groups (Fig. 2, Fig. 3A). Similarly, no specialized pro-resolving mediators, such as resolvins, protectins, or maresins, were detected (Fig. 2A and B & Fig 3A and B). Moreover, examining the potential differences in plasma PUFAs and oxylipins between UA patients and HC (Supplementary Fig. 3A–X) yielded no differences, suggesting that these two populations do not differ from each other in their PUFA- and oxylipin profile.

Most of the differences in oxylipins between aSAH at admission and both control groups were found in the higher abundance of HETEs. HETEs like 5-, 11-, 12- and 15-HETE can be further classified by their stereochemical configuration (*R-* or *S-*), depending on whether they are the product of either enzymatic action (*S*-enantiomer) or non-enzymatic auto-oxidation (racemic mixture) (Supplementary Fig. 4A). To provide insight into the biosynthesis of these differentially expressed HETEs in aSAH at admission, we determined the enantiomeric composition of 5-, 11-, 12- and 15-HETE in six plasma aliquots of previously analysed aSAH patients taken at admission. Chromatograms of the LC-MS/MS measurements of 5(*S*/*R*)-, 11(*S*/*R*)-, 12(*S*/*R*)- and 15 (*S*/*R*)-HETE from a representative patient are shown in Supplementary Fig. 4B. 5- and 12-HETE (*R/S* ratio ​= ​0.29) at admission predominantly exhibited *S*-configuration in aSAH patients, pointing towards the enzymatic synthesis of these oxylipins by 5(*S*)- and 12(*S*)-LOX respectively (Supplementary Fig. 4C and D) [28]. In contrast, 11- (*R/S* ratio ​= ​0.894) and 15-HETE (*R/S* ratio ​= ​0.897) were present in a racemic mixture, indicating that these oxylipins are the product of auto-oxidation [29].

### PUFAs and oxylipin levels in aSAH patients decrease 4 days after admission with the exception of 19,20-DiHDPA

**Fig. 4:**
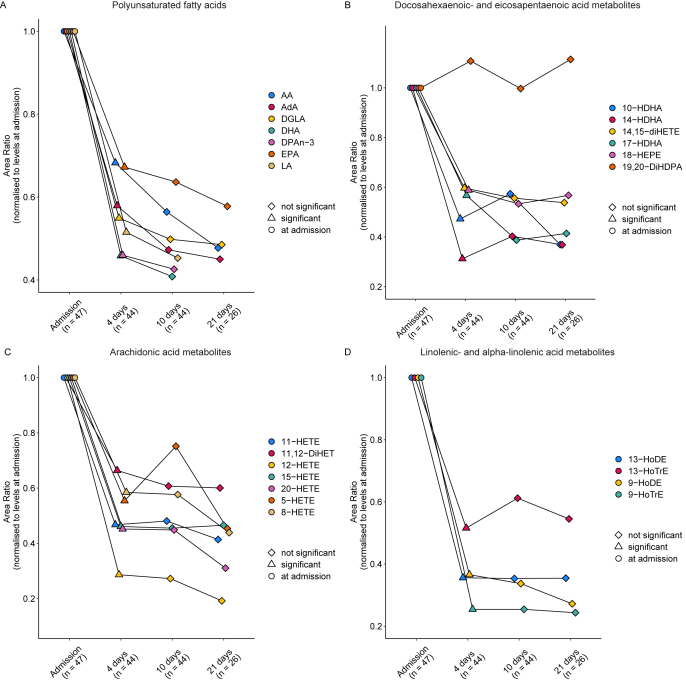
Longitudinal profile of polyunsaturated fatty acids (PUFAs) and oxylipins in aneurysmal subarachnoid haemorrhage (aSAH) patients. The shape of the data points corresponds to significance resulting from a paired Wilcoxon signed-rank test on non-normalised area ratio values and corrected for multiple testing with Benjamini-Hochberg procedure, comparing levels at each timepoint to the preceding timepoint. For visualisation the levels of the PUFAs and oxylipins were normalised to the levels at admission. A) PUFA levels at admission, day 4, day 10 and day 21. B) Docosahexaenoic- and eicosapentaenoic acid derived metabolite levels at admission, day 4, day 10 and day 21. C) Arachidonic acid derived metabolite levels at admission, day 4, day 10 and day 21. D) Linoleic- and alpha linoleic acid derived metabolite levels at admission, day 4, day 10 and day 21.

To understand whether and how PUFA and oxylipin levels change over time, plasma samples from aSAH patients were collected at admission and on days 4, 10, and 21 post-admission. These samples were subsequently measured using LC-MS/MS and compared across subsequent time points in a pairwise manner. The medians and IQR of all PUFAs and oxylipins, with respective details of the paired statistical tests are depicted in Supplementary Table 7. In general, we observed that all PUFAs revealed decreased levels at four days post-admission with no changes in their levels at any timepoint thereafter (Fig. 4A & Supplementary Fig. 5). Furthermore, all AA-, EPA-, DHA, LA- and ALA-derived oxylipins, except for 19,20-DiHDPA, showed a similar decrease at four days post-admission when compared to levels at admission in aSAH (Fig. 4B–D & Supplementary Fig. 5). Analogously, all oxylipins that showed an initial decrease at four days post-admission, did not show any changes in levels on the subsequent timepoints. Among these, 12-HETE exhibited the most pronounced decrease at day four after admission when compared to levels at admission. Although not significant, 5-HETE, 10-HDHA and 14-HDHA reveal slightly increased levels at 10 days, after an initial decrease at day four, followed by a decrement at 21 days (Fig. 4C and D). At 21 days post-admission levels of all PUFAs and oxylipins, except 19,20-DiHDPA, were found to remain lower than those measured at admission (Supplementary Table 8), suggesting that levels of PUFAs and oxylipins decreased after admission remain at low levels during disease progression. Of note, several patients were discharged (n ​= ​18) or died (n ​= ​3) before reaching the final timepoint. To ensure, the longitudinal changes in PUFA and oxylipin levels were not biased by the pattern of missingness, a sensitivity analysis was conducted. The longitudinal pattern in median levels of PUFAs and oxylipins and the results of the statistical testing were nearly identical at admission, day 4 and day 10 when patients were stratified for duration of hospitalization (<21 days vs. ​> ​21 days; Data not shown).

### PUFA and oxylipin levels are not associated with complications or outcome at any timepoint

To understand the potential of specific plasma PUFA and oxylipin levels to predict the development of DCI at a later stage, we applied various models like Random Forrest, Elastic Net Regression and Cox Proportional-Hazards Model. In addition to these analyses on the predictive qualities of PUFA- and oxylipin levels at admission, they were performed on levels measured on day 4, following removal of patients that already developed DCI. Due to the limited sample size, collinearity and high heterogeneity of DCI onset, these models did not yield significant results with respect to DCI. Next, we compared plasma PUFA and oxylipin levels of aSAH-DCI patients to aSAH-DCI patients at every timepoint. We did not observe any difference in PUFAs or oxylipins levels between aSAH ​+ ​DCI and aSAH-DCI patients at any timepoint (Supplementary Fig. 6 & Supplementary Table 9). Likewise, radiological DCI was not associated with any differences in PUFA and oxylipin levels at any timepoint when compared to patients without radiological DCI (data not shown). The sample size at day 21 was decreased due to either death (n ​= ​3) or discharge (aSAH+DCI n ​= ​8; aSAH-DCI n ​= ​10) before the last sampling timepoint. Contrasting the plasma PUFA and oxylipin levels between aSAH-DCI and aSAH ​+ ​DCI patients after stratification by duration of hospitalization (<21 days vs ​> ​21 days) revealed no differences at any timepoint (data not shown).

To identify a potential association between plasma NfL measured at admission, day 4 and day 10, and plasma levels of PUFAs and oxylipins at the respective timepoints, we applied a robust linear model with log-transformed values of NfL. Measurements of NfL where available for each timepoint of blood withdrawal for 46/47 aSAH patients. No association between plasma levels of NfL and plasma levels of PUFAs/oxylipins was observed at any timepoint after applying FDR correction. Next, we performed an ordinal logistic regression to investigate the putative association between PUFA- and oxylipin levels and the NIHSS on the day of blood withdrawal, as well as the mRS assessed at six months. Following FDR correction there were no associations between PUFA and oxylipin levels and either NIHSS or mRS. Before correction for FDR, we observed that few PUFAs and oxylipins showed an association with plasma levels of NfL, as well as the mRS and the NIHSS. We report these in Supplementary Table 10, as well as Supplementary Fig. 7A-H, 8A-G and 9A-F.

Neither the location of the aneurysm (Supplementary Table 11), treatment modality (Supplementary Table 12), modified Fisher scale on CT (Supplementary Table 13) nor WFNS grading at admission (Supplementary Table 14) showed any significant association with individual PUFA or oxylipin levels at admission.

## Discussion

This study investigated the plasma PUFA and oxylipin levels in aSAH patients and revealed elevated levels of all measured PUFAs and oxylipins at admission, compared to HC and those with UA. PUFA and oxylipin levels significantly declined by day four post-admission, except for continuously increased levels of 19,20-DiHDPA in aSAH patients. Levels of PUFAs and oxylipins did not differentiate patients who developed DCI.

Our findings extend previous findings from an untargeted LC-MS/MS approach, reporting generally increased levels of plasma PUFAs in aSAH patients, but not their specific composition [13]. Considering previous reports of increased LA, DHA and AA levels in the CSF of aSAH patients, but not in patients with other neurological diseases, such as meningitis or epilepsy, we speculate that the observed changes reflect aSAH-specific PUFA release [30]. In general, the brain contains higher levels of PUFAs than most other tissues, and astrocytes as well as endothelial cells are able to secrete PUFAs to maintain homeostatic neuronal function [31]. In an aSAH rodent model, higher levels of PUFAs within the brain parenchyma are measured quickly following haemorrhage, suggesting augmented local hydrolysis of PUFAs following aSAH [32]. We also show that increased plasma levels of PUFAs in aSAH patients are accompanied by higher levels of several AA-derived eicosanoids (i.e. 11,12-DiHET, 5-HETE, 8-HETE, 11-HETE, 12-HETE, 15-HETE and 20-HETE) and the DHA-derived metabolite 19,20-DiHDPA. Similarly, 5-, 11-, 12- and 20-HETE have been previously reported in CSF of aSAH patients [33,34]. The role of 20-HETE in aSAH pathology is well documented and higher levels of 20-HETE in the CSF of aSAH patients were predictive of poor outcome, likely resulting from its vasoconstrictive activity [16,18,35]. 12-HETE, on the other hand, is less well described in aSAH pathology and was found to be predominantly the product of oxygenation by 12S-LOX in this study. While 12-HETE is mostly found in activated platelets [28,36], it can also be produced by multiple other cell types (e.g. polymorphonuclear leukocytes [37] and astrocytes [38]). Previous studies show that 12-HETE can be both anti-thrombotic and pro-thrombotic, but its function is not completely understood [28]. When produced by endothelial cells, 12-HETE acts on the signalling molecule RhoA and its downstream effector PKCα, leading to enhanced endothelial intercellular adhesion molecule 1 (ICAM1) expression through nuclear factor-κB (NF-κB) activation [39]. This in turn leads to increased immune cell infiltration [39]. Considering the rapid release of 12-HETE following cerebral ischemia [40] and its increase in aSAH, it might present a promising candidate for pre-hospital diagnostics of aSAH. Moreover, the plethora of described effects of 12-HETE might make it a possible treatment target to modulate platelet aggregation, immune cell infiltration and micro-thrombi formation in the acute setting of aSAH. While 12(*S*)-HETE stood out as an interesting candidate, the chirality measurement was only done in a limited subset of patients. Therefore, these results must be interpreted with caution and future studies are warranted to validate the predominant *S*-configuration in the plasma of aSAH patients found in this study*.* Furhtermore, future studies are required to elucidate the exact origin and function of plasma PUFAs and oxylipins such as 12(*S*)-HETE in the context of the rupture of an aneurysm.

In our study, we also observed a decline of PUFA and oxylipin levels in the plasma of aSAH patients at 4 days compared to admission, which is also in accordance with previous findings in CSF [15,30,33]. However, both PUFA and certain oxylipin (i.e. 12- and 20-HETE) levels showed a second peak at 5 and 8–10 days post-admission, respectively, in CSF [30]. In contrast, in our study we found no increase in plasma PUFAs or oxylipin levels after day four. This discrepancy might suggest that neuroinflammatory processes in the later stages of aSAH may not be accurately reflected within the plasma of these patients. Interestingly, the exception was the soluble epoxide hydrolase (sEH) metabolite 19,20-DiHDPA, which was the only oxylipin exhibiting stably elevated levels throughout all timepoints in aSAH patients. Inhibition of sEH has been previously shown to act protective by preventing metabolization of epoxyeicosatrienoic acids (EETs) and epoxydocosapentaenoic acids (EpDPAs) in both acute ischemic stroke and diabetic retinopathy [41,42]. Particularly, in mice with diabetic retinopathy, the inhibition of sEH prevents a 19,20-DiHDPA-mediated increase in pericyte motility and migration into the extravascular space [42]. Previous work in human pulmonary arteries (HPA) showed that 19,20-EpDPE decreases the activation of RhoA and thereby leads to reduced Ca^2+^-sensitivity of HPA, which in turn causes a decrease of arterial tone induced by a thromboxane A_2_/prostanoid receptor agonist [43]. Furthermore, 19,20-EpDPE was demonstrated to reduce platelet aggregation *in vitro,* while platelets are known to be highly activated during aSAH [44,45]. The here observed high metabolization rates of 19,20-EpDPE to 19,20-DiHDPA might represent a non-homeostatic consequence of excessive CYP1A1/sEH-enzyme activity following aSAH. To clarify the exact role of 19,20-DiHDPA in aSAH, future studies should confirm its source and investigate its subsequent effect on the brain's vasculature.

Finally, we did not detect any differences in plasma PUFA and oxylipin levels associated with either clinical or radiological DCI. Therefore, based on PUFA and oxylipins levels at admission or at day four, it is not possible to distinguish which aSAH patients will develop DCI at a later stage. This study was powered to detect potential differences in clinical DCI but not radiological DCI. Accordingly, only very few patients presented with radiological DCI in this study. As a result, the power to detect differences in the plasma PUFA- and oxylipin levels associated with radiological DCI was likely insufficient. The difference in the occurrence of radiological DCI and clinical DCI in this study subpopulation highlights the complex nature of the diagnosis DCI. The differences in plasma PUFA and oxylipin levels observed in aSAH patients compared to controls in this study were limited to the acute setting. Therefore, it can be speculated that the standardized timepoints of blood collection do not capture the acute underlying processes during the onset of DCI. That is further complicated by the considerable heterogeneity in time of onset of DCI in this study, rendering it more difficult to pick up potential differences in plasma metabolites present in the acute moment of DCI. Plasma PUFA are quickly reacylated under physiological conditions [46,47], and their metabolites have predominately short half-lives [48]. Therefore, the standardized timepoints in wide intervals likely fail to capture the acute and short acting nature of the plasma *n-*3-and *n-*6-metabolism during the onset of DCI. Studies that report an association between DCI and oxylipins like 20-HETE or prostaglandins in CSF have typically obtained samples at shorter regular intervals after admission, likely better accounting for the high variability clinical diagnosis of DCI as well as the time of onset [49]. Moreover, in this study, we did not measure the levels of PUFAs and oxylipins between admission and day four. It could be possible that PUFA and oxylipin plasma levels in the 48 ​h following admission offer a more sensitive and clinically relevant tool to predict DCI at a later stage. Therefore, future studies investigating the potential changes in PUFA and oxylipin levels in plasma should consider blood collection at shorter time intervals following admission and subsequently at the time of the occurrence of DCI.

Several limitations of our study must be acknowledged. First, this study was performed in a European population, limiting its generalization to other ethnic backgrounds. Additionally, in this study cohort, the majority of the patients were women. While the higher number of women is in line with the global incidence of aSAH, it might limit the application of the results to aSAH patients generally [50]. Therefore, while aSAH is more prevalent in women, it is crucial that future studies aim for larger cohorts aiming for balanced numbers of women and men. Similarly, this study is limited by the sample size, which did not allow for inclusion of covariates in the regression models. Equivalently, the sample size was likely insufficient to detect associations between plasma levels of PUFAs and oxylipins and clinical outcome measures after FDR correction. As previously mentioned, we did not collect samples in the three days following admission, which might be a crucial time window to predict the development of DCI in aSAH patients based on differences in PUFA- and oxylipin levels. Furthermore, we did not detect an association between plasma NfL and PUFA- and oxlyipin levels at any timepoint. Plasma NfL was shown to be increased within 24h in aSAH patients, but reached the highest levels 7–12 days after rupture of the cerebral aneurysm [51]. The longitudinal trajectories of plasma PUFA and oxylipin levels were inverse to those of plasma NfL, likely accounting for the absence of association at admission. Additionally, the here reported sample size in combination with the nature of the collected data does not allow for the establishment of robust and clinical valuable multivariate predictive models, which is why we disregarded such an approach. Moreover, we determined the stereoisomeric configuration of a small subset of oxylipins in a limited number of patients. In the future, larger studies determining the stereoisomeric configuration would allow for more detailed inference about the origin of the detected lipid differences.

In conclusion, we here demonstrated that multiple PUFAs and oxylipins are affected in aSAH patients. A full understanding of the processes guiding damage and repair in aSAH is necessary to drive a better understanding of the pathology. It is apparent that the PUFA and oxylipin plasma signature might offer potential as a rapid blood marker in the pre-hospital setting to differentiate between aSAH and other intercranial pathologies, which would allow for quicker transport and access to adequate care for aSAH patients. Particularly, 12(*S*)-HETE stands out as an interesting candidate for diagnostic and therapeutic purposes. Furthermore, we demonstrate that 19,20-DiHDPA is chronically elevated in aSAH patients, possibly serving as a future treatment intervention. We did not detect PUFA- and oxylipin differences associated with the development of DCI. Future studies would need to determine the origin of the release of these oxylipins and compare the oxylipin plasma profiles of aSAH patients to other stroke subtypes and neurological pathologies that present in the emergency room.

## Author contributions

MF performed experiments, analysed data, and wrote the manuscript. MAT and DV provided material and valuable scientific input as well as revised the manuscript. MH performed experiments and provided valuable scientific input. SAR performed experiments. JC, WV, EvB, MG and HdV provided valuable scientific input as well as revised the manuscript. I.M and G.K supervised the study, conceived the design of the study, obtained funding, and revised the manuscript. All authors read and approved the publication of this manuscript.

## Acknowledgements

This study was funded by the European Union (ERC-2022-ADG, BRAIN, 101097983) and by Amsterdam Neuroscience (813294 to I.M. and G.K). Further support was provided by the Dutch Research Council (NWO Vidi Grant 91719305 to G.K.), and a grant from the Dutch Heart Association (03-006-2021-T019 to I.M.). Views and opinions expressed are, however, those of the author(s) only and do not necessarily reflect those of the European Union or the European Research Council. Neither the European Union nor the granting authority can be held responsible for them.

## Declaration of competing interest

The authors declare that they have no known competing financial interests or personal relationships that could have appeared to influence the work reported in this paper.
