# Supplementary material for: The polyunsaturated fatty acid and oxylipin plasma signature of aneurysmal subarachnoid haemorrhage, case-control study
Source: Neurotherapeutics. 2025 Sep 10;22(6):e00736. doi: 10.1016/j.neurot.2025.e00736 (PMC12664504; doi:10.1016/j.neurot.2025.e00736)
Supplement: Multimedia component 1 [file mmc1.pdf]

**Inclusion criteria**	**Exclusion criteria**
Admission within 24 hours after symptom	Traumatic subarachnoid haemorrhage
CT- or LP- confirmed subarachnoid haemorrhage	Patients with first blood collection >24 hours after SAH
Age 18 years and older	Platelet isolation >48 hours after blood collection
	Patients who do not speak either English or Dutch (also for patients with unruptured aneurysms)
	Hypercholesterimia
	Statin usage

Supplementary Table 1

Component	Q1 Mass	Q3 Mass	Retention Time	DP (Volts)	CE (volts)	CXP (Volts)
Leukotriene B4 d4	339,14	196,90	5,99	-70	-22	-19
15-HETE d8	327,18	226,00	6,82	-85	-18	-11
PGE2-d4	355,14	193,00	3,81	-50	-26	-17
8-iso-PGF2alpha-d4	357,30	197,00	3,45	-110	-34	-20
14(15)-EET-d11	330,20	219,00	7,01	-85	-15	-15
LXA4	351,10	114,80	4,37	-40	-20	-11
5-HETE	319,09	115,00	7,02	-65	-18	-11
8-HETE	319,08	154,90	6,92	-70	-20	-19
11-HETE	319,11	167,00	6,88	-70	-22	-15
12-HETE	319,09	179,00	6,92	-65	-20	-23
15-HETE	319,11	219,10	6,84	-55	-18	-9
15-HEPE	317,08	219,00	6,59	-65	-18	-19
18-HEPE	317,08	259,00	6,52	-5	-16	-7
LXB4	351,11	220,90	4,00	-60	-22	-13
Leukotriene B4	335,10	195,00	6,01	-65	-22	-21
17-HDHA	343,14	245,00	6,89	-65	-16	-15
14,15-diHETE	335,12	207,00	6,05	-65	-24	-21
19,20-DiHDPA	361,13	273,00	6,42	-55	-22	-15
RvE1	349,07	195,00	2,87	-95	-22	-13
RvE2	333,09	114,90	5,16	-35	-18	-15
18S-RvE3	333,07	245,20	5,68	-25	-16	-17
18R-RvE3	333,08	245,00	5,91	-55	-18	-23
TxB2	369,11	169,00	3,53	-55	-24	-15
6-trans-LTB4	335,12	194,90	5,76	-105	-22	-11
20-OH LTB4	351,11	195,00	3,05	-60	-24	-17
PGD2	351,12	233,00	3,92	-30	-16	-13
PDX	359,12	153,00	5,92	-70	-22	-9
PD1	359,12	153,00	5,98	-70	-22	-9
MaR1_2	359,20	250,20	5,98	-65	-20	-13
LTE4	438,10	333,10	6,08	-55	-26	-15
8S,15S-diHETE	335,10	207,90	5,76	-55	-22	-17
LTD4	495,15	177,00	5,76	-70	-28	-19
7,17-DiHDPA	361,12	198,90	6,07	-45	-26	-23
RvD1	375,13	215,00	4,46	-50	-26	-11
RvD2	375,13	277,10	4,14	-60	-18	-15
6t,12epi-LTB4	335,11	194,90	5,86	-80	-22	-25
PGF2a	353,11	193,00	4,10	-80	-34	-11
PGE2_2	351,20	271,10	3,83	-50	-22	-21
17-OH-DH-HETE	347,12	247,00	7,22	-110	-22	-27
13-HoTrE	292,98	195,00	6,48	-45	-24	-19

13-HoDE	295,02	194,90	6,75	-110	-24	-21
7S-MaR1	359,07	249,90	5,71	-20	-20	-19
15-Keto-PGE2	349,04	234,90	3,50	-65	-20	-13
13,14dihydro-15keto-PGF2a	353,08	195,00	4,39	-110	-32	-11
8-iso-PGE2	351,07	271,00	3,63	-5	-24	-19
8-iso-PGF2a	353,09	193,00	3,46	-135	-34	-11
9-HoTrE	293,00	170,90	6,44	-75	-20	-15
9-HoDE	295,04	171,00	6,75	-130	-22	-7
AA	303,04	205,10	7,75	-155	-20	-11
DHA	327,06	229,20	7,74	-115	-18	-11
EPA	301,03	202,90	7,56	-125	-18	-21
AdA	331,11	233,00	7,94	-130	-22	-11
DPAn-3	329,09	231,10	7,83	-50	-20	-17
DHAd5	332,00	288,10	7,74	-75	-16	-13
LA	279,04	261,00	7,76	-115	-28	-13
ALA/GLA	277,04	233,00	7,56	-90	-22	-29
PGJ2	333,03	271,00	5,12	-30	-22	-17
5,15-diHETE	335,04	173,10	5,87	-55	-20	-11
10-HDHA	343,07	153,00	6,93	-25	-20	-15
7-HDHA	343,09	141,10	6,98	-85	-18	-23
5F3T IsoP	351,02	114,80	2,80	-95	-26	-13
14(15)EET	319,01	218,90	7,03	-5	-16	-55
11(12)EET	318,95	166,90	7,13	-90	-18	-19
8(9)EET	319,01	154,90	7,15	-60	-18	-13
15Deoxy PGJ2	315,01	203,00	6,34	-50	-28	-19
4F4T NP	377,05	270,90	4,20	-15	-26	-13
17F2 Dihomo IsoP	381,10	318,90	5,05	-115	-32	-41
20-HETE_2	319,05	289,10	6,68	-70	-24	-15
4-HDHA	343,08	101,00	7,11	-50	-18	-9
14(S)-HDHA	343,06	204,90	6,93	-60	-18	-27
5-HEPE	317,05	114,90	6,72	-55	-18	-11
12-HEPE	317,04	179,00	6,65	-60	-18	-17
5-KETE	317,04	203,10	7,05	-70	-24	-11
12-KETE	317,05	153,00	6,86	-60	-22	-9
15-KETE	317,03	113,00	6,77	-10	-22	-5
19(20)-EpDPA	343,07	281,10	7,05	-70	-16	-11
DGLA	305,09	261,20	7,86	-85	-22	-13

Supplementary Table 2

SAH			
Timepoint	Component Name	Median (ng/ml)	IQR
Admission	11-HETE	0,63	0,10
Admission	12-HETE	1,67	0,96
Admission	14,15-diHETE	0,74	0,32
Admission	15-HETE	0,84	0,13
Admission	18-HEPE	0,61	0,08
Admission	5-HETE	0,76	0,29
Admission	8-HETE	0,55	0,08
Admission	AA	1719,39	1267,06
Admission	AdA	206,49	140,01
Admission	DHA	2366,64	1200,67
Admission	DPAn-3	838,26	747,91
Admission	EPA	258,11	234,98
Admission	LA	40705,48	22998,49
Day 4	11-HETE	0,54	0,08
Day 4	12-HETE	0,80	0,39
Day 4	14,15-diHETE	0,55	0,24
Day 4	15-HETE	0,65	0,15
Day 4	18-HEPE	0,56	0,06
Day 4	5-HETE	0,64	0,26
Day 4	8-HETE	0,49	0,08
Day 4	AA	1166,74	1208,03
Day 4	AdA	129,31	154,52
Day 4	DHA	1104,11	974,88
Day 4	DPAn-3	389,65	413,79
Day 4	EPA	177,80	229,41
Day 4	LA	21059,15	13560,29
Day 10	11-HETE	0,54	0,04
Day 10	12-HETE	0,78	0,26
Day 10	14,15-diHETE	0,52	0,17
Day 10	15-HETE	0,66	0,11
Day 10	18-HEPE	0,55	0,05
Day 10	5-HETE	0,70	0,14
Day 10	8-HETE	0,49	0,03
Day 10	AA	939,15	945,81
Day 10	AdA	103,19	109,78
Day 10	DHA	1037,04	971,86
Day 10	DPAn-3	346,80	251,24
Day 10	EPA	170,63	185,49
Day 10	LA	18388,82	12331,97
Day 21	11-HETE	0,53	0,06
Day 21	12-HETE	0,68	0,65
Day 21	14,15-diHETE	0,53	0,18

Day 21	15-HETE	0,65	0,14
Day 21	18-HEPE	0,56	0,06
Day 21	5-HETE	0,61	0,09
Day 21	8-HETE	0,47	0,04
Day 21	AA	809,56	600,30
Day 21	AdA	98,57	69,81
Day 21	DHA	890,28	717,96
Day 21	DPAn-3	259,99	161,03
Day 21	EPA	153,89	110,99
Day 21	LA	16052,22	10134,88
**Control**			
	**Component Name**	**Median (ng/ml)**	**IQR**
	11-HETE	0,57	0,04
	12-HETE	0,80	0,16
	14,15-diHETE	0,55	0,09
	15-HETE	0,70	0,11
	18-HEPE	0,54	0,05
	5-HETE	0,68	0,10
	8-HETE	0,52	0,05
	AA	1388,99	755,58
	AdA	115,83	82,05
	DHA	1489,66	1162,12
	DPAn-3	488,00	313,85
	EPA	145,25	100,53
	LA	29824,32	12572,74
**Unruptured**			
	**Component Name**	**Median (ng/ml)**	**IQR**
	11-HETE	0,55	0,06
	12-HETE	0,78	0,21
	14,15-diHETE	0,57	0,15
	15-HETE	0,71	0,12
	18-HEPE	0,59	0,08
	5-HETE	0,67	0,14
	8-HETE	0,51	0,07
	AA	1133,61	755,78
	AdA	96,71	112,70
	DHA	1469,41	1529,50
	DPAn-3	374,27	504,93
	EPA	204,21	159,79
	LA	21541,71	18506,78

Supplementary Table 3

Component	Q1 Mass	Q3 Mass	Retention Time	DP (Volts)	CE (Volts)	CXP (Volts)
5-HETE	319.083	115.018	4.600	-55,0	-18,0	-33,0
11-HETE	319.083	167.000	4.550	-70,0	-22,0	-15,0
12-HETE	319.083	179.113	5.060	-55,0	-20,0	-49,0
15-HETE	319.083	219.168	4.970	-55,0	-18,0	-17,0
20-HETE	319.083	289.228	3.900	-100,0	-24,0	-15,0

Supplementary Table 4

Timepoint	Component Name	ß	exp(ß)	StdError	t value	Lower95	Upper95	p	p adj.
T0	10-HDHA	0,02	1,02	0,10	0,21	-0,17	0,21	0,84	1
T0	11,12-DiHET	-0,03	0,97	0,13	-0,25	-0,29	0,22	0,81	1
T0	11-HETE	0,01	1,01	0,05	0,18	-0,08	0,10	0,86	1
T0	12-HETE	0,00	1,00	0,00	-0,47	-0,01	0,00	0,64	1
T0	13-HoDE	0,00	1,00	0,00	-0,58	-0,01	0,00	0,56	1
T0	13-HoTrE	0,00	1,00	0,03	0,00	-0,06	0,06	1,00	1
T0	14-HDHA	0,00	1,00	0,01	-0,37	-0,02	0,01	0,71	1
T0	14,15-diHETE	0,04	1,05	0,28	0,16	-0,50	0,59	0,88	1
T0	15-HETE	-0,01	0,99	0,04	-0,31	-0,10	0,07	0,76	1
T0	17-HDHA	0,11	1,12	0,09	1,27	-0,06	0,29	0,21	1
T0	18-HEPE	0,14	1,15	0,17	0,79	-0,20	0,48	0,43	1
T0	19,20-DiHDPA	0,00	1,00	0,03	0,05	-0,06	0,07	0,96	1
T0	20-HETE	0,03	1,03	0,04	0,71	-0,05	0,10	0,48	1
T0	5-HETE	0,01	1,01	0,09	0,12	-0,16	0,19	0,90	1
T0	8-HETE	0,03	1,03	0,18	0,19	-0,32	0,39	0,85	1
T0	9-HoDE	-0,01	0,99	0,01	-0,77	-0,02	0,01	0,44	1
T0	9-HoTrE	-0,05	0,95	0,10	-0,57	-0,24	0,13	0,57	1
T0	AA	0,00	1,00	0,02	0,23	-0,04	0,05	0,82	1
T0	AdA	0,03	1,03	0,07	0,43	-0,10	0,16	0,67	1
T0	DGLA	0,05	1,05	0,05	1,05	-0,04	0,14	0,30	1
T0	DHA	0,00	1,00	0,00	0,82	0,00	0,01	0,42	1
T0	DPAn-3	0,02	1,02	0,02	1,55	-0,01	0,05	0,13	1
T0	EPA	0,01	1,01	0,01	0,72	-0,02	0,03	0,48	1
T0	LA	0,00	1,00	0,00	-0,09	-0,01	0,01	0,93	1
T1	10-HDHA	0,62	1,87	0,48	1,30	-0,32	1,57	0,20	1
T1	11,12-DiHET	0,17	1,18	0,24	0,71	-0,29	0,63	0,48	1
T1	11-HETE	0,06	1,06	0,08	0,75	-0,10	0,22	0,46	1
T1	12-HETE	-0,01	0,99	0,02	-0,61	-0,06	0,03	0,54	1
T1	13-HoDE	-0,02	0,98	0,01	-1,73	-0,04	0,00	0,09	1
T1	13-HoTrE	-0,15	0,86	0,21	-0,75	-0,56	0,25	0,46	1
T1	14-HDHA	-0,14	0,87	0,19	-0,74	-0,51	0,23	0,46	1
T1	14,15-diHETE	0,38	1,47	0,52	0,73	-0,64	1,41	0,47	1
T1	15-HETE	0,06	1,06	0,10	0,54	-0,15	0,26	0,60	1
T1	17-HDHA	0,31	1,37	0,22	1,40	-0,13	0,75	0,17	1
T1	18-HEPE	0,59	1,81	0,42	1,39	-0,24	1,42	0,17	1
T1	19,20-DiHDPA	0,06	1,06	0,04	1,59	-0,01	0,14	0,12	1
T1	20-HETE	-0,03	0,97	0,08	-0,35	-0,20	0,14	0,73	1
T1	5-HETE	0,07	1,07	0,09	0,73	-0,11	0,25	0,47	1
T1	8-HETE	-0,02	0,98	0,31	-0,06	-0,63	0,59	0,95	1
T1	9-HoDE	-0,02	0,98	0,02	-0,86	-0,06	0,02	0,39	1
T1	9-HoTrE	-0,33	0,72	0,44	-0,74	-1,20	0,54	0,46	1
T1	AA	0,01	1,01	0,02	0,59	-0,03	0,06	0,56	1
T1	AdA	0,09	1,09	0,11	0,83	-0,12	0,29	0,41	1
T1	DGLA	0,07	1,07	0,07	0,91	-0,08	0,21	0,37	1

T1	DHA	0,01	1,01	0,01	1,11	-0,01	0,03	0,27	1
T1	DPAn-3	0,04	1,04	0,03	1,40	-0,02	0,10	0,17	1
T1	EPA	0,04	1,04	0,03	1,46	-0,01	0,09	0,15	1
T1	LA	0,00	1,00	0,01	-0,06	-0,01	0,01	0,95	1
T2	10-HDHA	-0,32	0,72	0,36	-0,91	-1,02	0,37	0,37	1
T2	11,12-DiHET	-0,10	0,90	0,26	-0,39	-0,61	0,40	0,70	1
T2	11-HETE	-0,11	0,90	0,11	-0,94	-0,33	0,12	0,36	1
T2	12-HETE	-0,04	0,96	0,03	-1,32	-0,11	0,02	0,19	1
T2	13-HoDE	-0,01	0,99	0,01	-1,42	-0,03	0,00	0,16	1
T2	13-HoTrE	-0,04	0,96	0,15	-0,30	-0,33	0,24	0,77	1
T2	14-HDHA	-0,09	0,91	0,20	-0,46	-0,49	0,30	0,65	1
T2	14,15-diHETE	-0,17	0,84	0,41	-0,41	-0,98	0,64	0,68	1
T2	15-HETE	-0,07	0,94	0,15	-0,44	-0,36	0,23	0,66	1
T2	17-HDHA	0,33	1,40	0,34	0,97	-0,34	1,01	0,34	1
T2	18-HEPE	-0,22	0,81	0,29	-0,75	-0,78	0,35	0,46	1
T2	19,20-DiHDPA	0,04	1,04	0,04	0,95	-0,04	0,11	0,35	1
T2	20-HETE	-0,07	0,93	0,13	-0,54	-0,31	0,18	0,59	1
T2	5-HETE	0,02	1,02	0,18	0,13	-0,34	0,39	0,90	1
T2	8-HETE	-0,24	0,79	0,37	-0,64	-0,97	0,49	0,53	1
T2	9-HoDE	-0,02	0,98	0,01	-1,60	-0,05	0,00	0,12	1
T2	9-HoTrE	-0,29	0,75	0,25	-1,17	-0,77	0,19	0,25	1
T2	AA	0,00	1,00	0,02	-0,21	-0,05	0,04	0,84	1
T2	AdA	-0,05	0,95	0,10	-0,52	-0,24	0,14	0,61	1
T2	DGLA	-0,04	0,96	0,06	-0,71	-0,17	0,08	0,48	1
T2	DHA	-0,01	0,99	0,01	-1,08	-0,02	0,01	0,29	1
T2	DPAn-3	-0,01	0,99	0,02	-0,63	-0,06	0,03	0,53	1
T2	EPA	0,01	1,01	0,02	0,42	-0,03	0,05	0,68	1
T2	LA	0,00	1,00	0,01	-0,64	-0,02	0,01	0,53	1
T3	10-HDHA	0,27	1,31	0,97	0,28	-1,62	2,16	0,78	1
T3	11,12-DiHET	-0,58	0,56	0,38	-1,54	-1,32	0,16	0,14	1
T3	11-HETE	-0,05	0,95	0,13	-0,38	-0,32	0,21	0,71	1
T3	12-HETE	-0,08	0,92	0,04	-2,25	-0,16	-0,01	0,03	1
T3	13-HoDE	0,01	1,01	0,03	0,19	-0,06	0,07	0,85	1
T3	13-HoTrE	0,41	1,50	0,35	1,17	-0,28	1,09	0,26	1
T3	14-HDHA	-0,65	0,52	0,35	-1,84	-1,35	0,04	0,08	1
T3	14,15-diHETE	-0,48	0,62	1,01	-0,47	-2,46	1,51	0,64	1
T3	15-HETE	-0,16	0,85	0,23	-0,67	-0,62	0,30	0,51	1
T3	17-HDHA	0,25	1,28	0,73	0,33	-1,19	1,69	0,74	1
T3	18-HEPE	-0,70	0,50	0,82	-0,85	-2,30	0,91	0,40	1
T3	19,20-DiHDPA	-0,04	0,96	0,05	-0,81	-0,15	0,06	0,42	1
T3	20-HETE	-0,36	0,70	0,38	-0,95	-1,10	0,38	0,35	1
T3	5-HETE	0,00	1,00	0,32	0,00	-0,62	0,63	1,00	1
T3	8-HETE	-0,53	0,59	0,73	-0,72	-1,96	0,91	0,48	1
T3	9-HoDE	-0,04	0,96	0,07	-0,65	-0,18	0,09	0,52	1
T3	9-HoTrE	-0,02	0,98	1,03	-0,02	-2,04	2,01	0,99	1

T3	AA	-0,03	0,97	0,04	-0,62	-0,11	0,06	0,54	1
T3	AdA	-0,26	0,77	0,23	-1,13	-0,72	0,20	0,27	1
T3	DGLA	-0,24	0,78	0,13	-1,95	-0,49	0,00	0,06	1
T3	DHA	-0,01	0,99	0,01	-0,56	-0,03	0,02	0,58	1
T3	DPAn-3	-0,04	0,96	0,06	-0,71	-0,15	0,07	0,49	1
T3	EPA	-0,04	0,96	0,05	-0,69	-0,14	0,07	0,50	1
T3	LA	-0,01	0,99	0,01	-0,38	-0,03	0,02	0,71	1

Supplementary Table 5

Group	Component	Median	IQR				
SAH	10-HDHA	0,20	0,20				
SAH	11,12-DiHET	0,56	0,33				
SAH	11-HETE	1,37	0,75				
SAH	12-HETE	3,32	2,60				
SAH	13-HoDE	12,06	8,48				
SAH	13-HoTrE	0,44	0,27				
SAH	14,15-diHETE	0,23	0,15				
SAH	14-HDHA	0,32	0,47				
SAH	15-HETE	1,16	0,42				
SAH	17-HDHA	0,24	0,25				
SAH	18-HEPE	0,30	0,19				
SAH	19,20-DiHDPA	1,54	1,32				
SAH	20-HETE	1,03	1,02				
SAH	5-HETE	0,49	0,50				
SAH	8-HETE	0,33	0,18				
SAH	9-HoDE	7,11	4,90				
SAH	9-HoTrE	0,36	0,28				
SAH	AA	4,23	3,06				
SAH	AdA	0,70	0,48				
SAH	DGLA	1,51	1,12				
SAH	DHA	16,94	8,60				
SAH	DPAn-3	3,52	3,07				
SAH	EPA	2,76	2,47	**SAH vs UA**			
SAH	LA	25,22	14,22	**Foldchange**	**W**	**p**	**p adj,**
UA	10-HDHA	0,16	0,11	1,21	653	0,284	0,3408
UA	11,12-DiHET	0,39	0,20	1,90	889	< 0.0001	0,00035
UA	11-HETE	0,72	0,49	1,39	765	0,0141	0,0302
UA	12-HETE	0,91	0,56	3,64	955	< 0.0001	< 0.0001
UA	13-HoDE	10,14	8,57	1,19	647	0,318	0,36343
UA	13-HoTrE	0,43	0,25	1,04	580	0,852	0,852
UA	14,15-diHETE	0,16	0,08	1,96	722	0,0551	0,08816
UA	14-HDHA	0,16	0,22	1,44	716	0,0653	0,09795
UA	15-HETE	0,72	0,40	1,60	831	0,00095	0,00325
UA	17-HDHA	0,19	0,17	1,22	696	0,11	0,14667
UA	18-HEPE	0,25	0,17	1,23	664	0,228	0,288
UA	19,20-DiHDPA	1,13	0,79	1,37	763	0,0151	0,0302
UA	20-HETE	0,44	0,29	2,35	950	< 0.0001	< 0.0001
UA	5-HETE	0,34	0,25	1,44	771	0,0114	0,02736
UA	8-HETE	0,25	0,15	1,35	756	0,0192	0,03545
UA	9-HoDE	5,67	5,50	1,25	705	0,0877	0,12381
UA	9-HoTrE	0,35	0,36	1,00	600	0,668	0,69704
UA	AA	2,82	1,82	1,50	751	0,0226	0,03874
UA	AdA	0,33	0,38	2,14	848	0,00041	0,00199
UA	DGLA	0,78	0,69	1,94	827	0,00114	0,00342

UA	DHA	10,52	10,95	1,61	771	0,0114	0,02736
UA	DPAn-3	1,62	2,07	2,18	837	0,00071	0,00285
UA	EPA	2,20	1,68	1,26	629	0,436	0,47564
UA	LA	13,36	11,44	1,89	882	< 0.0001	0,00039
				**SAH vs HC**			
				Foldchange	W	**p**	**p adj.**
HC	10-HDHA	0,13	0,09	1,47	190	0,038	0,05067
HC	11,12-DiHET	0,38	0,27	1,60	101	0,00011	0,0009
HC	11-HETE	0,86	0,27	1,43	180	0,0236	0,04046
HC	12-HETE	0,96	0,50	3,45	60	< 0.0001	< 0.0001
HC	13-HoDE	7,11	12,01	1,70	249	0,319	0,33287
HC	13-HoTrE	0,36	0,09	1,24	216	0,111	0,12686
HC	14,15-diHETE	0,15	0,05	2,05	166	0,0114	0,02487
HC	14-HDHA	0,15	0,17	1,58	182	0,0261	0,04176
HC	15-HETE	0,69	0,43	1,67	94	< 0.0001	0,00071
HC	17-HDHA	0,14	0,17	1,65	174	0,0174	0,03212
HC	18-HEPE	0,15	0,14	2,05	162	0,00911	0,02448
HC	19,20-DiHDPA	0,96	0,59	1,61	186	0,0316	0,0474
HC	20-HETE	0,48	0,36	2,13	121	0,0006	0,0036
HC	5-HETE	0,33	0,16	1,48	163	0,00964	0,02448
HC	8-HETE	0,27	0,12	1,23	172	0,0157	0,0314
HC	9-HoDE	6,03	7,24	1,18	261	0,434	0,434
HC	9-HoTrE	0,28	0,19	1,28	226	0,158	0,17236
HC	AA	3,36	2,24	1,26	211	0,0919	0,11028
HC	AdA	0,38	0,34	1,85	150	0,00447	0,01788
HC	DGLA	0,89	0,44	1,70	164	0,0102	0,02448
HC	DHA	10,36	8,16	1,64	188	0,0347	0,04899
HC	DPAn-3	1,95	1,44	1,81	161	0,0086	0,02448
HC	EPA	1,58	1,03	1,75	202	0,0641	0,08097
HC	LA	18,24	10,27	1,38	130	0,00117	0,00562

Supplementary Table 6

Timepoint	Component Name	Median	IQR			
Admission	10-HDHA	0,20	0,20			
Admission	11,12-DiHET	0,56	0,33			
Admission	11-HETE	1,37	0,75			
Admission	12-HETE	3,32	2,60			
Admission	13-HoDE	12,06	8,48			
Admission	13-HoTrE	0,44	0,27			
Admission	14,15-diHETE	0,23	0,15			
Admission	14-HDHA	0,32	0,47			
Admission	15-HETE	1,16	0,42			
Admission	17-HDHA	0,24	0,25			
Admission	18-HEPE	0,30	0,19			
Admission	19,20-DiHDPA	1,54	1,32			
Admission	20-HETE	1,03	1,02			
Admission	5-HETE	0,49	0,50			
Admission	8-HETE	0,33	0,18			
Admission	9-HoDE	7,11	4,90			
Admission	9-HoTrE	0,36	0,28			
Admission	AA	4,23	3,06			
Admission	AdA	0,70	0,48			
Admission	DGLA	1,51	1,12			
Admission	DHA	16,94	8,60			
Admission	DPAn-3	3,52	3,07			
Admission	EPA	2,76	2,47	**Comparing to admission**		
Admission	LA	25,22	14,22	**W**	**p**	**p adj.**
Day 4	10-HDHA	0,09	0,08	849	< 0.0001	< 0.0001
Day 4	11,12-DiHET	0,36	0,20	829	< 0.0001	< 0.0001
Day 4	11-HETE	0,67	0,60	908	< 0.0001	< 0.0001
Day 4	12-HETE	0,97	1,07	887	< 0.0001	< 0.0001
Day 4	13-HoDE	4,38	3,19	958	< 0.0001	< 0.0001
Day 4	13-HoTrE	0,25	0,11	886	< 0.0001	< 0.0001
Day 4	14,15-diHETE	0,15	0,12	757	0,00177	0,00202
Day 4	14-HDHA	0,11	0,13	871	< 0.0001	< 0.0001
Day 4	15-HETE	0,55	0,49	901	< 0.0001	< 0.0001
Day 4	17-HDHA	0,14	0,12	853	< 0.0001	< 0.0001
Day 4	18-HEPE	0,18	0,14	853	< 0.0001	< 0.0001
Day 4	19,20-DiHDPA	1,77	1,83	414	0.35100	0.35100
Day 4	20-HETE	0,47	0,49	838	< 0.0001	< 0.0001
Day 4	5-HETE	0,28	0,44	765	0.00124	0.00157
Day 4	8-HETE	0,19	0,17	831	< 0.0001	< 0.0001
Day 4	9-HoDE	2,62	2,13	963	< 0.0001	< 0.0001
Day 4	9-HoTrE	0,09	0,13	958	< 0.0001	< 0.0001
Day 4	AA	2,90	2,92	692	0,0208	0.02269

Day 4	AdA	0,44	0,53	760	0.00155	0.00186
Day 4	DGLA	0,88	0,79	807	0,00015	0,0002
Day 4	DHA	7,90	6,98	917	< 0.0001	< 0.0001
Day 4	DPAn-3	1,68	1,70	870	< 0.0001	< 0.0001
Day 4	EPA	1,92	2,41	676	0,0342	0.03569
Day 4	LA	13,07	8,39	926	< 0.0001	< 0.0001
				**Comparing to levels at day 4 post admission**		
Timepoint	Component Name	Median	IQR	**W**	**p**	**p adj.**
Day 10	10-HDHA	0,12	0,08	372	0,327	1
Day 10	11,12-DiHET	0,33	0,15	458	0,941	1
Day 10	11-HETE	0,68	0,32	458	0,941	1
Day 10	12-HETE	0,93	0,69	462	0,902	1
Day 10	13-HoDE	4,46	3,89	408	0,594	1
Day 10	13-HoTrE	0,30	0,22	323	0,11	1
Day 10	14,15-diHETE	0,13	0,09	541	0,269	1
Day 10	14-HDHA	0,15	0,15	400	0,528	1
Day 10	15-HETE	0,56	0,35	501	0,544	1
Day 10	17-HDHA	0,10	0,13	560	0,179	1
Day 10	18-HEPE	0,16	0,13	477	0,757	1
Day 10	19,20-DiHDPA	1,54	1,16	512	0,457	1
Day 10	20-HETE	0,48	0,38	502	0,536	1
Day 10	5-HETE	0,39	0,23	417	0,674	1
Day 10	8-HETE	0,20	0,06	452	1	1
Day 10	9-HoDE	2,48	1,91	450	0,99	1
Day 10	9-HoTrE	0,10	0,13	399	0,519	1
Day 10	AA	2,35	2,28	504	0,519	1
Day 10	AdA	0,35	0,37	512	0,457	1
Day 10	DGLA	0,77	0,55	483	0,701	1
Day 10	DHA	7,42	6,96	463	0,892	1
Day 10	DPAn-3	1,50	1,03	490	0,638	1
Day 10	EPA	1,84	1,95	464	0,882	1
Day 10	LA			497	0,577	1
				**Comparing to levels at day 10 post admission**		
Timepoint	Component Name	Median	IQR	**W**	**p**	**p adj.**
Day 21	10-HDHA	0,07	0,08	245	0,0796	0,4776
Day 21	11,12-DiHET	0,32	0,25	167	0,842	0,9193
Day 21	11-HETE	0,59	0,44	202	0,515	0,9193
Day 21	12-HETE	0,64	1,75	193	0,671	0,9193
Day 21	13-HoDE	4,32	2,60	195	0,635	0,9193
Day 21	13-HoTrE	0,27	0,19	212	0,367	0,9193
Day 21	14,15-diHETE	0,13	0,09	160	0,708	0,9193
Day 21	14-HDHA	0,13	0,11	206	0,452	0,9193
Day 21	15-HETE	0,56	0,45	184	0,842	0,9193
Day 21	17-HDHA	0,11	0,11	196	0,617	0,9193

Day 21	18-HEPE	0,19	0,14	165	0,803	0,9193
Day 21	19,20-DiHDPA	1,74	1,58	126	0,217	0,9193
Day 21	20-HETE	0,32	0,27	246	0,0751	0,4776
Day 21	5-HETE	0,23	0,16	258	0,0357	0,4284
Day 21	8-HETE	0,14	0,07	258	0,0357	0,4284
Day 21	9-HoDE	1,96	1,24	207	0,437	0,9193
Day 21	9-HoTrE	0,09	0,07	179	0,94	0,94
Day 21	AA	2,03	1,45	182	0,881	0,9193
Day 21	AdA	0,34	0,24	198	0,582	0,9193
Day 21	DGLA	0,76	0,46	150	0,532	0,9193
Day 21	DHA	6,37	5,14	183	0,861	0,9193
Day 21	DPAn-3	1,15	0,66	192	0,689	0,9193
Day 21	EPA	1,67	1,17	146	0,468	0,9193
Day 21	LA	9,97	6,27	188	0,764	0,9193

Supplementary Table 7

Component Name	W	p	p adj.
10-HDHA	326,00	< 0.0001	< 0.0001
11,12-DiHET	311,00	0,00025	0,00033
11-HETE	330,00	< 0.0001	< 0.0001
12-HETE	314,00	0,00017	0,00025
13-HoDE	351,00	< 0.0001	< 0.0001
13-HoTrE	292,00	0,00218	0,00249
14,15-diHETE	279,00	0,00729	0,00795
14-HDHA	310,00	0,00028	0,00036
15-HETE	339,00	< 0.0001	< 0.0001
17-HDHA	337,00	< 0.0001	< 0.0001
18-HEPE	294,00	0,00178	0,00214
19,20-DiHDPA	150,00	0,532	0,532
20-HETE	345,00	< 0.0001	< 0.0001
5-HETE	326,00	< 0.0001	< 0.0001
8-HETE	343,00	< 0.0001	< 0.0001
9-HoDE	351,00	< 0.0001	< 0.0001
9-HoTrE	345,00	< 0.0001	< 0.0001
AA	311,00	0,00025	0,00033
AdA	327,00	< 0.0001	< 0.0001
DGLA	321,00	< 0.0001	< 0.0001
DHA	346,00	< 0.0001	< 0.0001
DPAn-3	344,00	< 0.0001	< 0.0001
EPA	273,00	0,012	0,01252
LA	343,00	< 0.0001	< 0.0001

Supplementary Table 8

Component Name	Timepoint	W	p	p adj.
10-HDHA	Admission	222	0,49	1,00
11,12-DiHET	Admission	226	0,55	1,00
11-HETE	Admission	181	0,11	1,00
12-HETE	Admission	208	0,32	1,00
13-HoDE	Admission	219	0,45	1,00
13-HoTrE	Admission	186	0,13	1,00
14,15-diHETE	Admission	280	0,55	1,00
14-HDHA	Admission	230	0,61	1,00
15-HETE	Admission	240	0,78	1,00
17-HDHA	Admission	251	0,97	1,00
18-HEPE	Admission	213	0,37	1,00
19,20-DiHDPA	Admission	269	0,73	1,00
20-HETE	Admission	228	0,58	1,00
5-HETE	Admission	205	0,28	1,00
8-HETE	Admission	161	0,04	1,00
9-HoDE	Admission	197	0,21	1,00
9-HoTrE	Admission	172	0,07	1,00
AA	Admission	272	0,68	1,00
AdA	Admission	209	0,33	1,00
DGLA	Admission	237	0,73	1,00
DHA	Admission	262	0,85	1,00
DPAn-3	Admission	225	0,54	1,00
EPA	Admission	257	0,94	1,00
LA	Admission	277	0,60	1,00
10-HDHA	Day 4	216	0,73	1,00
11,12-DiHET	Day 4	204	0,52	1,00
11-HETE	Day 4	184	0,26	1,00
12-HETE	Day 4	228	0,95	1,00
13-HoDE	Day 4	208	0,59	1,00
13-HoTrE	Day 4	201	0,48	1,00
14,15-diHETE	Day 4	237	0,90	1,00
14-HDHA	Day 4	240	0,84	1,00
15-HETE	Day 4	208	0,59	1,00
17-HDHA	Day 4	239	0,86	1,00
18-HEPE	Day 4	200	0,46	1,00
19,20-DiHDPA	Day 4	204	0,52	1,00
20-HETE	Day 4	211	0,64	1,00
5-HETE	Day 4	188	0,31	1,00
8-HETE	Day 4	185	0,27	1,00
9-HoDE	Day 4	192	0,35	1,00
9-HoTrE	Day 4	197	0,42	1,00
AA	Day 4	182	0,24	1,00
AdA	Day 4	194	0,38	1,00

DGLA	Day 4	182	0,24	1,00
DHA	Day 4	214	0,69	1,00
DPAn-3	Day 4	198	0,43	1,00
EPA	Day 4	183	0,25	1,00
LA	Day 4	212	0,66	1,00
10-HDHA	Day 10	244	0,57	1,00
11,12-DiHET	Day 10	227	0,88	1,00
11-HETE	Day 10	217	0,94	1,00
12-HETE	Day 10	270	0,22	1,00
13-HoDE	Day 10	100	0,00	0,19
13-HoTrE	Day 10	164	0,16	1,00
14,15-diHETE	Day 10	289	0,09	1,00
14-HDHA	Day 10	266	0,26	1,00
15-HETE	Day 10	242	0,60	1,00
17-HDHA	Day 10	220	1,00	1,00
18-HEPE	Day 10	260	0,33	1,00
19,20-DiHDPA	Day 10	261	0,32	1,00
20-HETE	Day 10	237	0,69	1,00
5-HETE	Day 10	191	0,47	1,00
8-HETE	Day 10	223	0,96	1,00
9-HoDE	Day 10	140	0,04	1,00
9-HoTrE	Day 10	143	0,05	1,00
AA	Day 10	217	0,94	1,00
AdA	Day 10	242	0,60	1,00
DGLA	Day 10	245	0,55	1,00
DHA	Day 10	283	0,12	1,00
DPAn-3	Day 10	265	0,27	1,00
EPA	Day 10	274	0,18	1,00
LA	Day 10	232	0,78	1,00
10-HDHA	Day 21	81	0,89	1,00
11,12-DiHET	Day 21	91	0,50	1,00
11-HETE	Day 21	70	0,69	1,00
12-HETE	Day 21	77	0,98	1,00
13-HoDE	Day 21	82	0,85	1,00
13-HoTrE	Day 21	76	0,94	1,00
14,15-diHETE	Day 21	98	0,30	1,00
14-HDHA	Day 21	73	0,81	1,00
15-HETE	Day 21	85	0,73	1,00
17-HDHA	Day 21	86	0,69	1,00
18-HEPE	Day 21	88	0,61	1,00
19,20-DiHDPA	Day 21	83	0,81	1,00
20-HETE	Day 21	53	0,19	1,00
5-HETE	Day 21	86	0,69	1,00
8-HETE	Day 21	76	0,94	1,00
9-HoDE	Day 21	78	1,00	1,00

9-HoTrE	Day 21	85	0,73	1,00
AA	Day 21	91	0,50	1,00
AdA	Day 21	80	0,94	1,00
DGLA	Day 21	88	0,61	1,00
DHA	Day 21	78	1,00	1,00
DPAn-3	Day 21	72	0,77	1,00
EPA	Day 21	96	0,35	1,00
LA	Day 21	80	0,94	1,00

Supplementary Table 9

Variable	Component	Timepoint	Est.	exp(Est.)	StdError	t	Lower95	Upper95	p	p adj.
NFL	13-HoDE	Day 4	-0,12	0,89	0,05	-2,65	-0,2118	-0,0316	0,012	0,261
NFL	13-HoTrE	Day 4	-1,62	0,20	0,79	-2,06	-3,1583	-0,0754	0,046	0,700
NFL	9-HoDE	Day 4	-0,26	0,77	0,08	-3,40	-0,4060	-0,1090	0,002	0,077
NFL	9-HoTrE	Day 4	-5,07	0,01	1,51	-3,36	-8,0264	-2,1169	0,002	0,077
NFL	AA	Day 4	-0,22	0,81	0,07	-2,95	-0,3588	-0,0724	0,005	0,159
NFL	LA	Day 4	-0,04	0,96	0,02	-2,24	-0,0787	-0,0052	0,031	0,557
NFL	AA	Day 10	-0,34	0,71	0,12	-2,79	-0,5809	-0,1013	0,008	0,362
NFL	LA	Day 10	-0,09	0,91	0,03	-2,81	-0,1584	-0,0283	0,008	0,362

Variable	Component	Timepoint	Est.	OR	Wald Z		Lower95	Upper95	p	p adj.
NIHSS	11,12-DiHET	Day 4	-5,06	0,01	-2,36		0,0001	0,4262	0,018	0,834
NIHSS	AA	Day 4	-0,51	0,60	-2,55		0,4073	0,8894	0,011	0,834
NIHSS	20-HETE	Day 10	-3,01	0,05	-2,27		0,0036	0,6653	0,023	0,353
NIHSS	5-HETE	Day 10	-4,63	0,01	-2,34		0,0002	0,4706	0,019	0,353
NIHSS	AA	Day 10	-0,75	0,47	-2,94		0,2866	0,7789	0,003	0,299
NIHSS	AdA	Day 10	-3,39	0,03	-2,44		0,0022	0,5162	0,015	0,353
NIHSS	DGLA	Day 10	-2,11	0,12	-2,36		0,0209	0,6989	0,018	0,353
NIHSS	DPAn-3	Day 10	-0,87	0,42	-2,30		0,1979	0,8793	0,022	0,353

Variable	Component	Timepoint	Est.	OR	Wald Z		Lower95	Upper95	p	p adj.
mRS	11,12-DiHET	Day 4	-3,93	0,02	-2,31		0,0007	0,5544	0,021	0,875
mRS	13-HoDE	Day 4	-0,26	0,77	-2,64		0,6322	0,9349	0,008	0,761
mRS	9-HoDE	Day 4	-0,40	0,67	-2,18		0,4714	0,9612	0,029	0,875
mRS	9-HoTrE	Day 4	-7,18	0,00	-2,07		0,0000	0,6869	0,039	0,875
mRS	AA	Day 10	-0,38	0,68	-2,35		0,4960	0,9388	0,019	0,933
mRS	LA	Day 10	-0,11	0,90	-2,32		0,8206	0,9837	0,021	0,933

Supplementary Table 10

Component Name	W	DF	p	p adj.
5-HETE	17,7	6	0,01	0,17
11-HETE	11,6	6	0,07	0,43
10-HDHA	11,5	6	0,07	0,43
8-HETE	10,9	6	0,09	0,43
20-HETE	9,9	6	0,13	0,43
15-HETE	9,7	6	0,14	0,43
13-HoTrE	9,6	6	0,14	0,43
14-HDHA	9,5	6	0,15	0,43
18-HEPE	9,1	6	0,17	0,43
17-HDHA	8,8	6	0,19	0,43
DPAn-3	8,3	6	0,22	0,43
12-HETE	8,1	6	0,23	0,43
DHA	7,9	6	0,25	0,43
11,12-DiHET	7,8	6	0,25	0,43
19,20-DiHDPA	7,4	6	0,29	0,46
LA	6,7	6	0,35	0,52
AdA	6,1	6	0,42	0,58
9-HoDE	5,9	6	0,44	0,58
EPA	5,5	6	0,48	0,61
DGLA	5,1	6	0,53	0,63
AA	4,5	6	0,61	0,69
13-HoDE	3,1	6	0,80	0,87
14,15-diHETE	2,4	6	0,88	0,89
9-HoTrE	2,3	6	0,89	0,89

Supplementary Table 11

Component Name	W	DF	p	p adj.
AA	10,01	4	0,04	0,73
DGLA	9,02	4	0,06	0,73
AdA	7,79	4	0,10	0,80
DPAn-3	6,53	4	0,16	0,98
EPA	3,68	4	0,45	0,98
13-HoTrE	3,42	4	0,49	0,98
9-HoTrE	3,25	4	0,52	0,98
18-HEPE	3,16	4	0,53	0,98
5-HETE	3,02	4	0,55	0,98
20-HETE	2,36	4	0,67	0,98
15-HETE	2,35	4	0,67	0,98
DHA	2,14	4	0,71	0,98
8-HETE	1,80	4	0,77	0,98
17-HDHA	1,58	4	0,81	0,98
LA	1,49	4	0,83	0,98
11,12-DiHET	1,24	4	0,87	0,98
11-HETE	1,21	4	0,88	0,98
14-HDHA	1,17	4	0,88	0,98
13-HoDE	1,07	4	0,90	0,98
10-HDHA	1,04	4	0,90	0,98
9-HoDE	0,92	4	0,92	0,98
12-HETE	0,66	4	0,96	0,98
14,15-diHETE	0,56	4	0,97	0,98
19,20-DiHDPA	0,45	4	0,98	0,98

Supplementary Table 12

Component Name	W	DF	p	p adj.
10-HDHA	6,22	2	0,04	0,76
17-HDHA	5,03	2	0,08	0,76
14-HDHA	4,13	2	0,13	0,76
18-HEPE	3,74	2	0,15	0,76
11,12-DiHET	3,48	2	0,18	0,76
19,20-DiHDPA	3,33	2	0,19	0,76
DHA	2,62	2	0,27	0,84
DPAn-3	1,86	2	0,39	0,84
LA	1,81	2	0,40	0,84
9-HoTrE	1,65	2	0,44	0,84
5-HETE	1,57	2	0,46	0,84
EPA	1,51	2	0,47	0,84
8-HETE	1,14	2	0,57	0,84
12-HETE	1,02	2	0,60	0,84
9-HoDE	0,99	2	0,61	0,84
14,15-diHETE	0,95	2	0,62	0,84
20-HETE	0,91	2	0,63	0,84
DGLA	0,90	2	0,64	0,84
13-HoDE	0,81	2	0,67	0,84
AdA	0,72	2	0,70	0,84
13-HoTrE	0,59	2	0,74	0,85
15-HETE	0,50	2	0,78	0,85
AA	0,28	2	0,87	0,91
11-HETE	0,15	2	0,93	0,93

Supplementary Table 13

Component Name	Coeffecient	Odds ratio	SE	t value	p value
10-HDHA	0,00	1,00	0,239	0,00	1,00
11,12-DiHET	0,49	1,64	0,559	0,88	0,38
11-HETE	0,07	1,07	0,515	0,13	0,90
12-HETE	0,25	1,28	0,292	0,86	0,39
13-HoDE	-0,15	0,86	0,521	-0,28	0,78
13-HoTrE	0,29	1,33	0,416	0,69	0,49
14-HDHA	0,21	1,24	0,223	0,96	0,33
14,15-diHETE	0,19	1,21	0,453	0,42	0,67
15-HETE	-0,13	0,88	0,564	-0,23	0,82
17-HDHA	0,09	1,10	0,280	0,34	0,74
18-HEPE	0,14	1,15	0,346	0,41	0,68
19,20-DiHDPA	0,83	2,30	0,410	2,03	0,04
20-HETE	-0,01	0,99	0,374	-0,02	0,98
5-HETE	-0,23	0,79	0,417	-0,56	0,57
8-HETE	0,08	1,08	0,447	0,18	0,86
9-HoDE	-0,25	0,78	0,524	-0,47	0,64
9-HoTrE	-0,53	0,59	0,374	-1,43	0,15
AA	-0,56	0,57	0,619	-0,90	0,37
AdA	-0,28	0,75	0,493	-0,57	0,57
DGLA	-0,42	0,66	0,529	-0,79	0,43
DHA	-0,10	0,91	0,489	-0,20	0,84
DPAn-3	-0,02	0,98	0,459	-0,03	0,97
EPA	-0,25	0,78	0,337	-0,75	0,45
LA	0,30	1,35	0,643	0,47	0,64

Supplementary Table 14
