# Supplementary material for: The polyunsaturated fatty acid and oxylipin plasma signature of aneurysmal subarachnoid haemorrhage, case-control study
Source: Neurotherapeutics. 2025 Sep 10;22(6):e00736. doi: 10.1016/j.neurot.2025.e00736 (PMC12664504; doi:10.1016/j.neurot.2025.e00736)
Supplement: Multimedia component 2 [file mmc2.pdf]

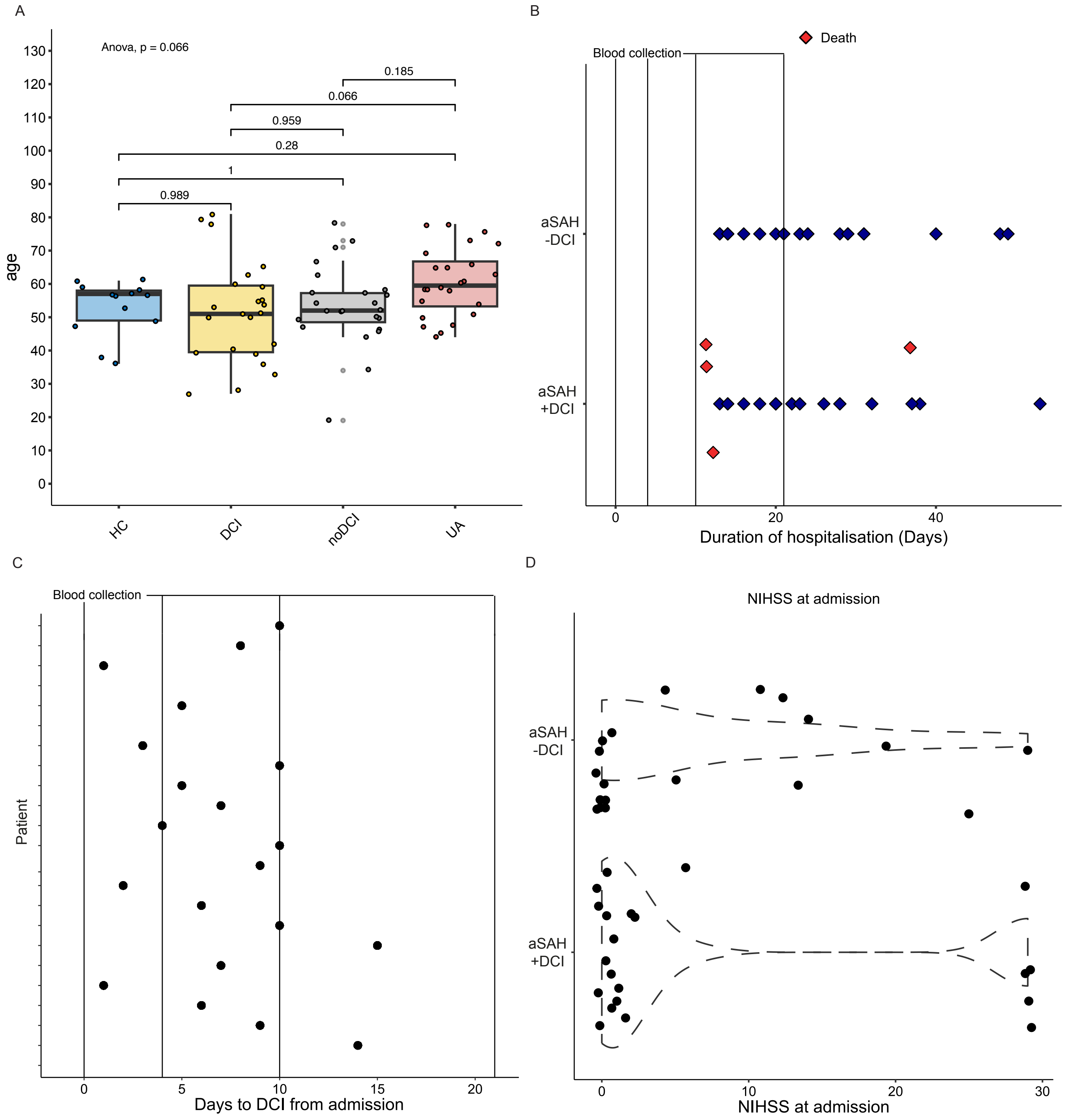

Supplementary Figure 1

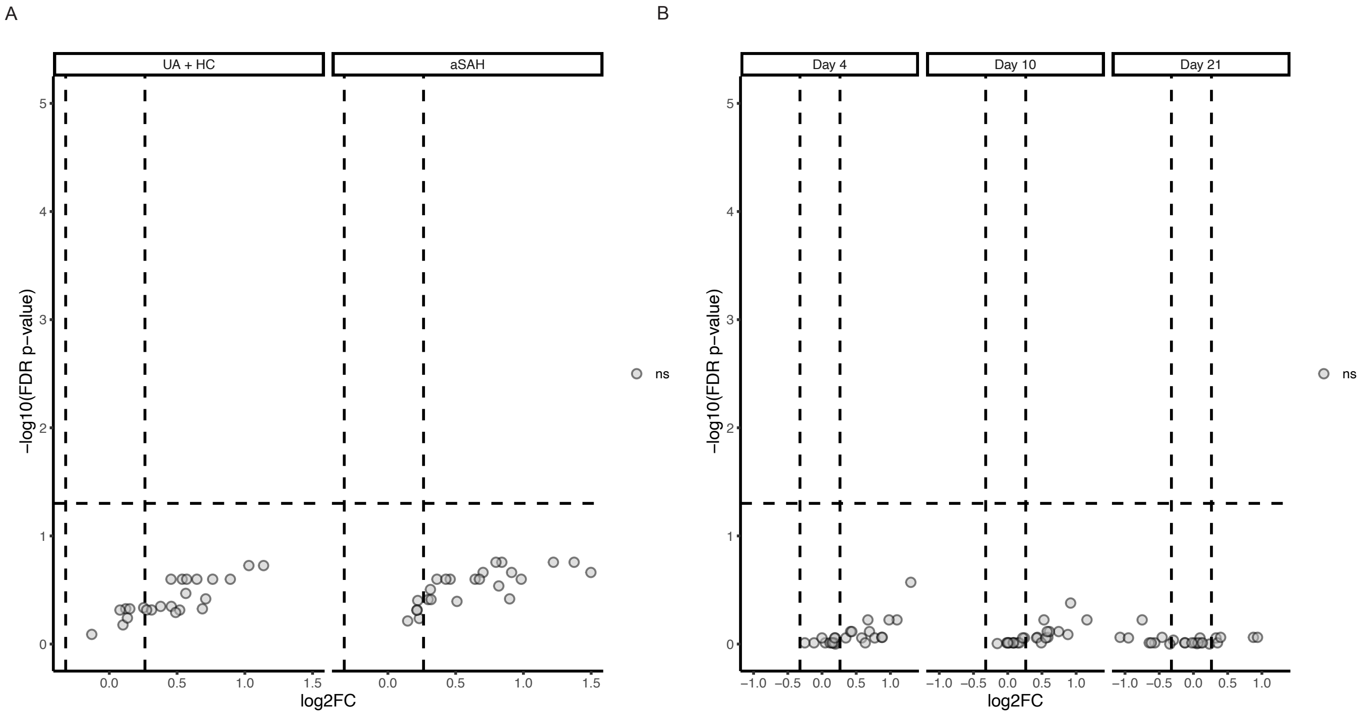

Supplementary Figure 2

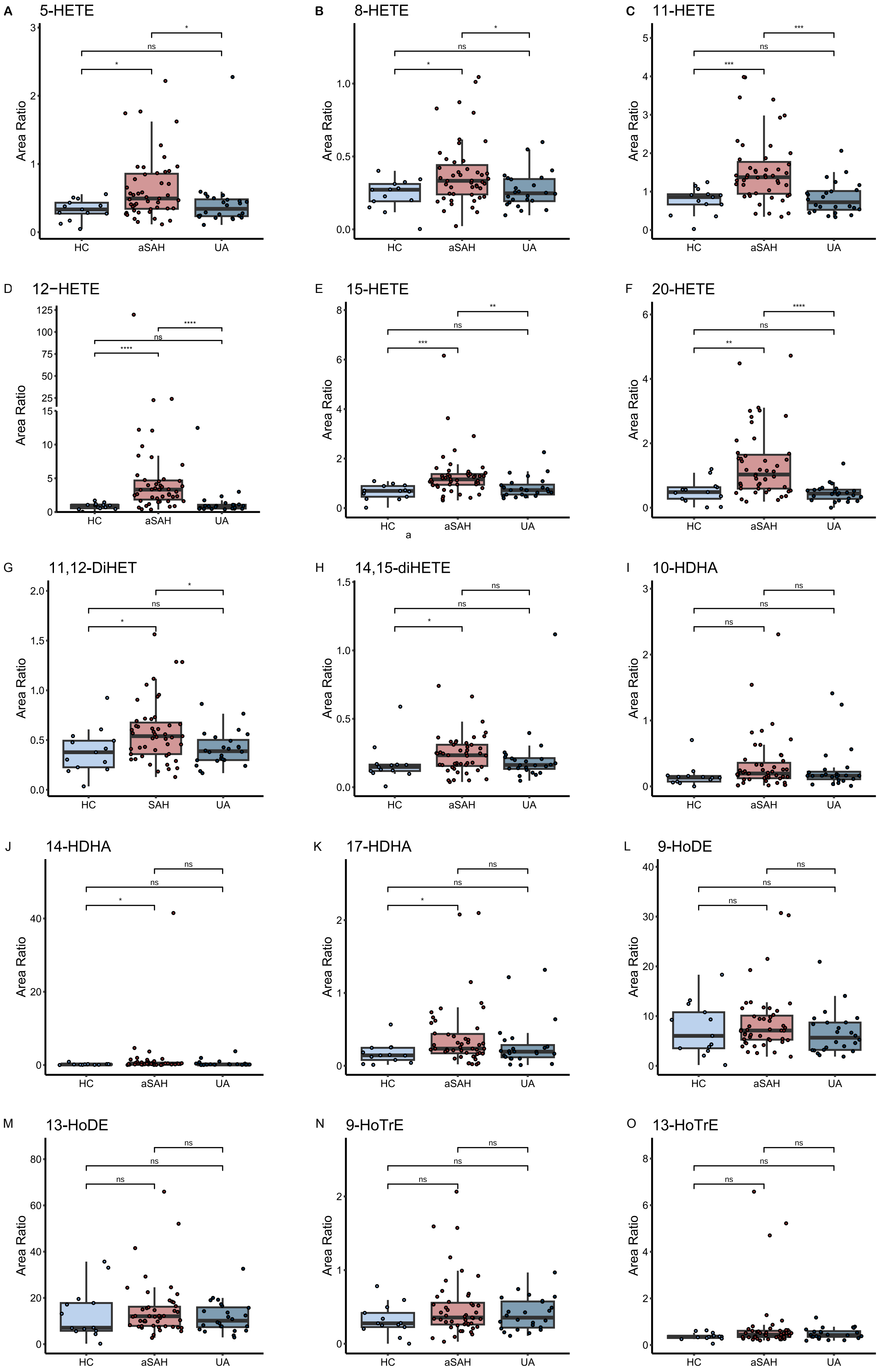
A 5-HETE
B 8-HETE
C 11-HETE
D 12−HETE
E 15-HETE
F 20-HETE
G 11,12-DiHET
H 14,15-diHETE
I 10-HDHA
J 14-HDHA
K 17-HDHA
L 9-HoDE
M 13-HoDE
N 9-HoTrE
O 13-HoTrE
Area Ratio
HC
aSAH
SAH
UA
a
ns
*
**
***
****

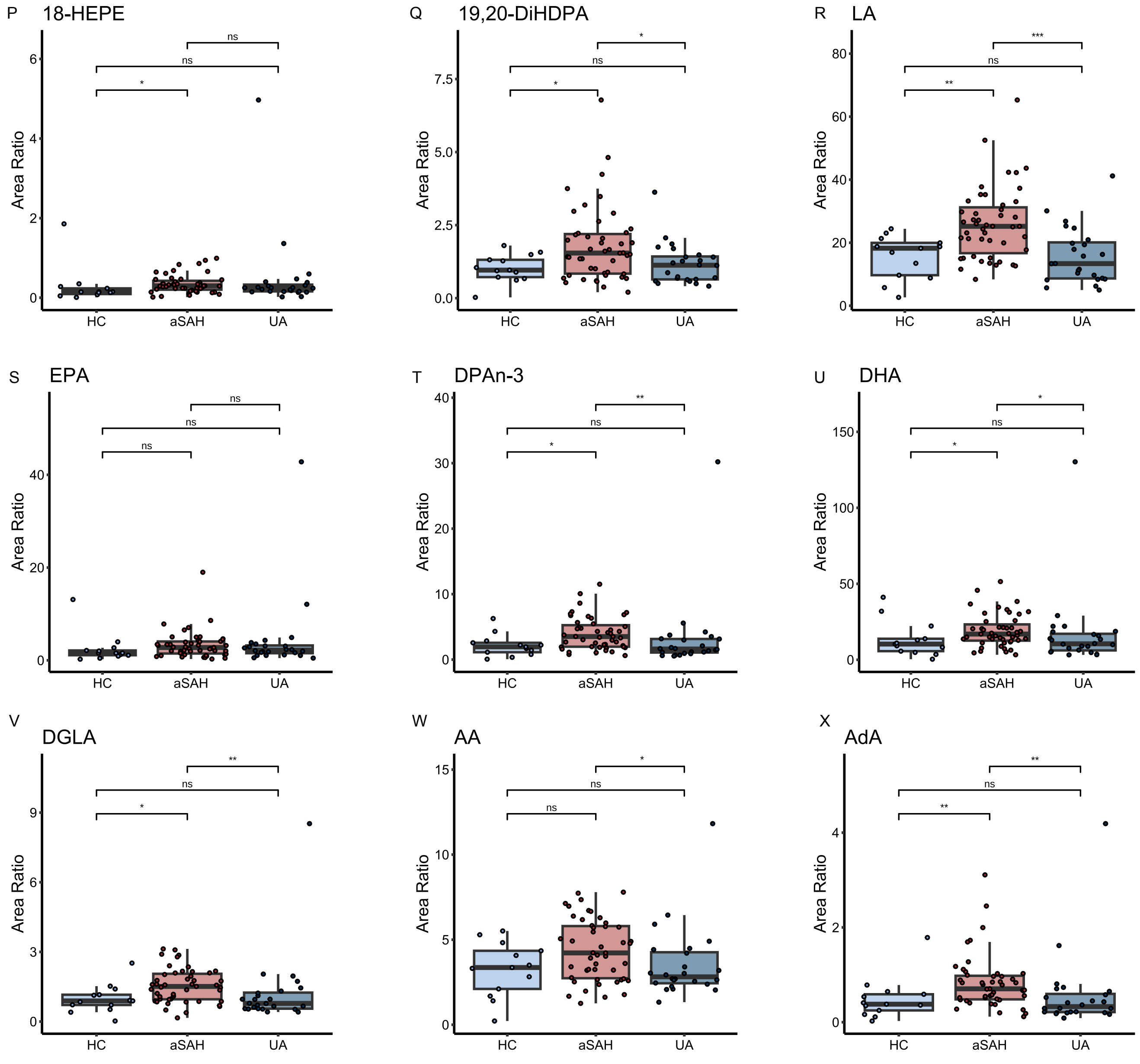

Supplementary Figure 3A - X

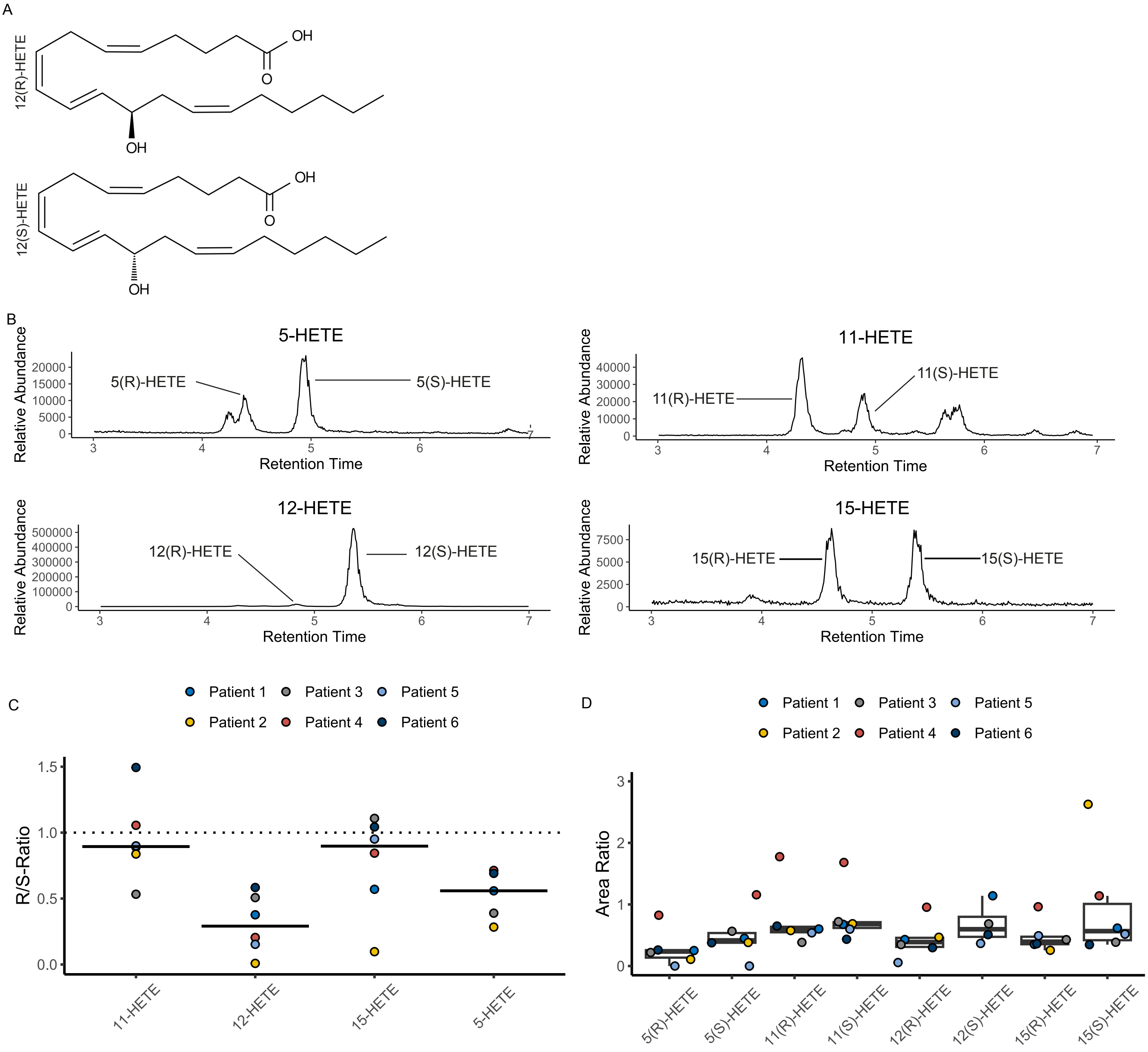

Supplementary Figure 4

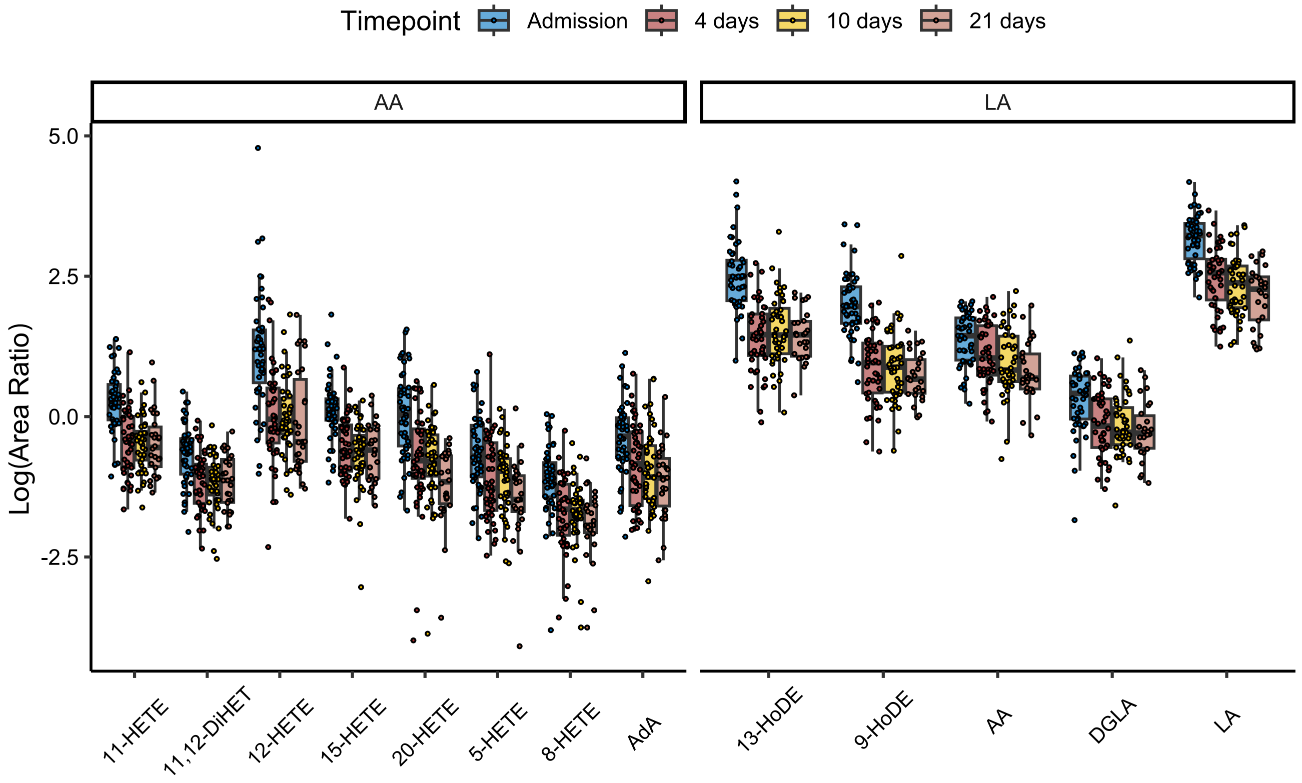

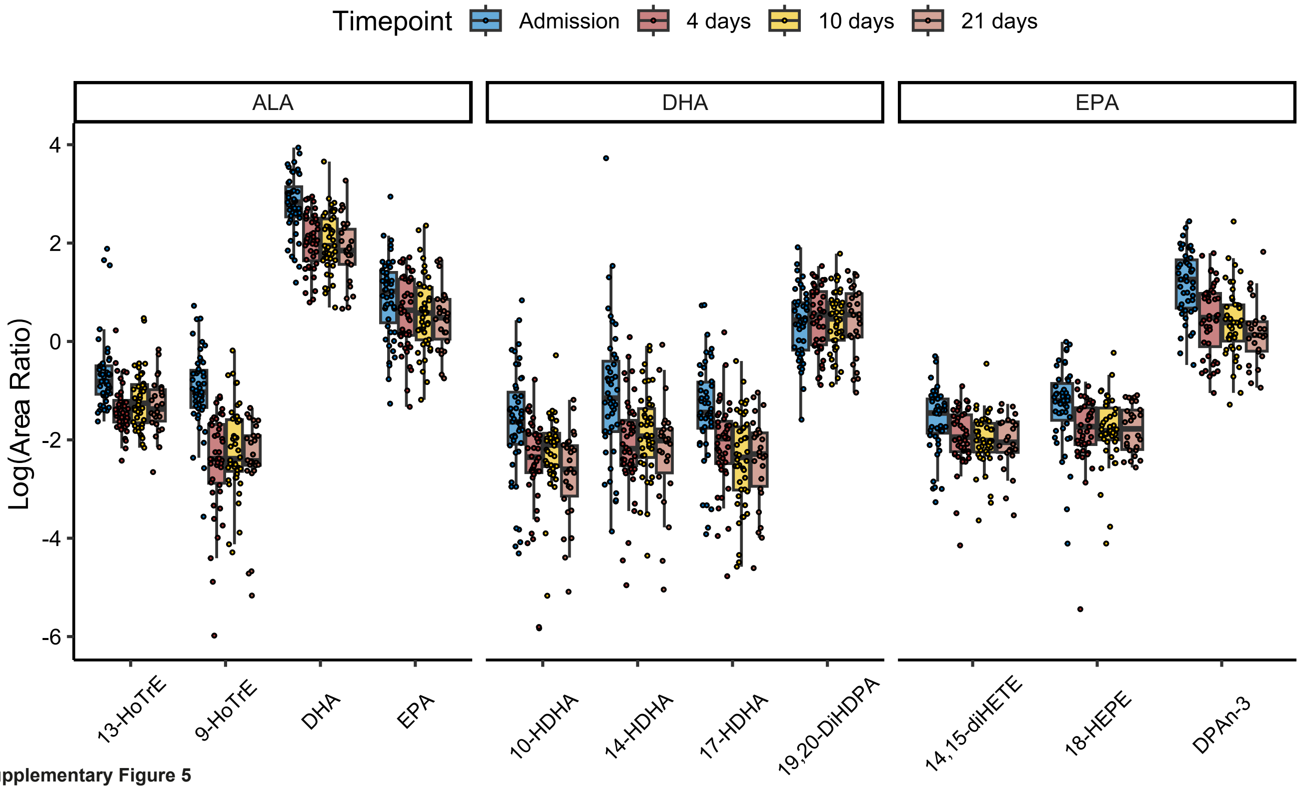

Supplementary Figure 5

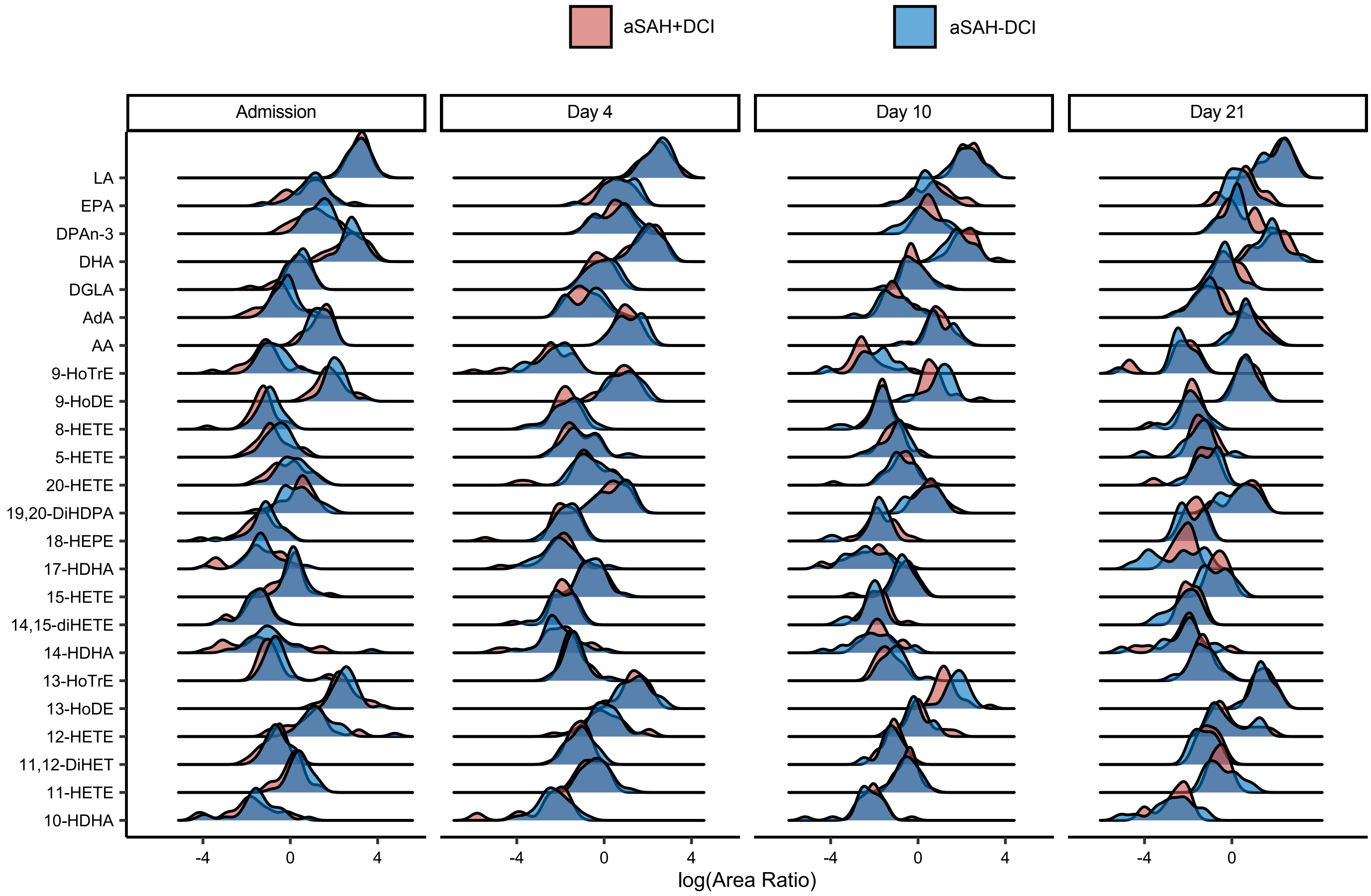

Supplementary Figure 6

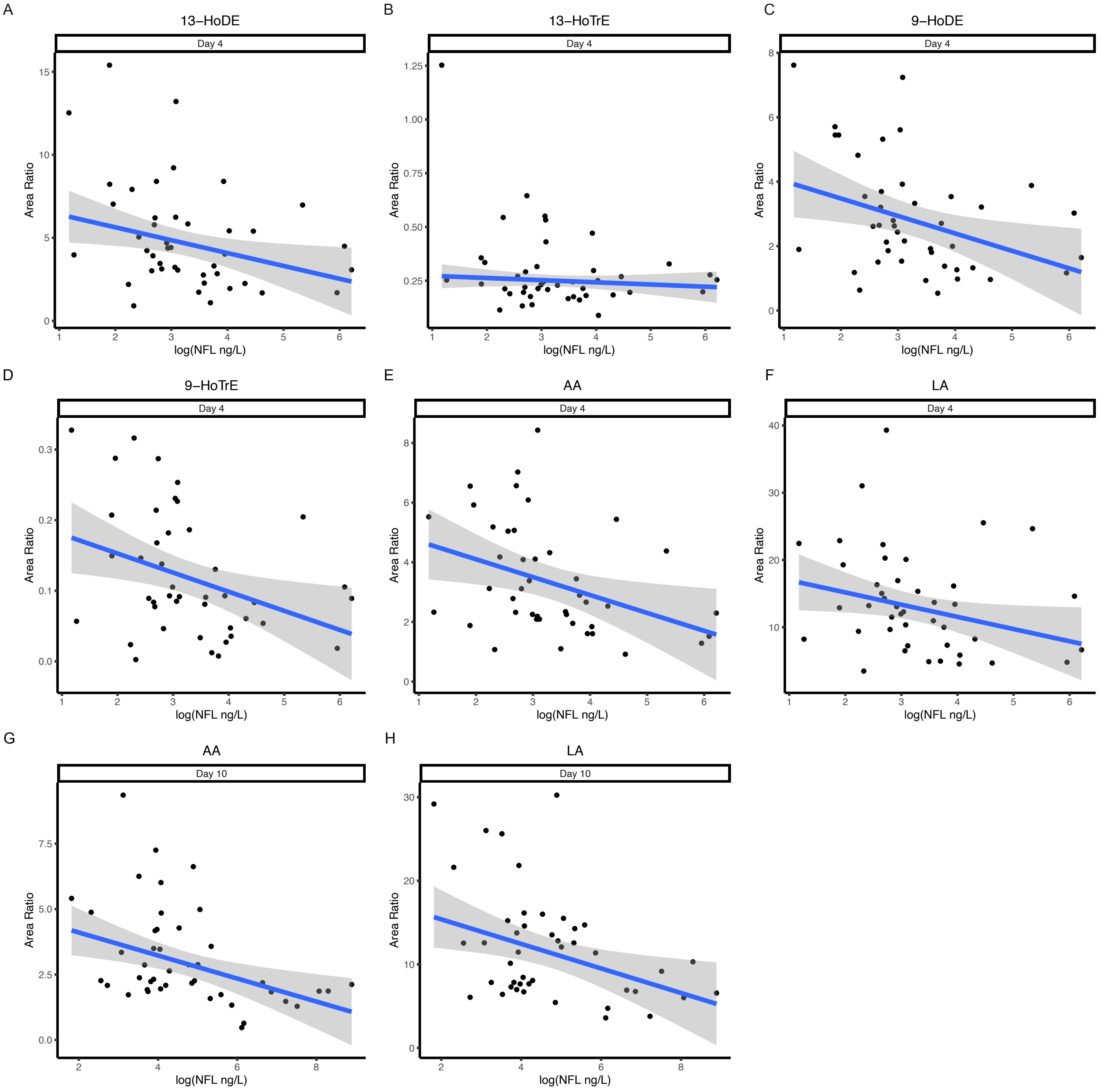

Supplementary Figure 7

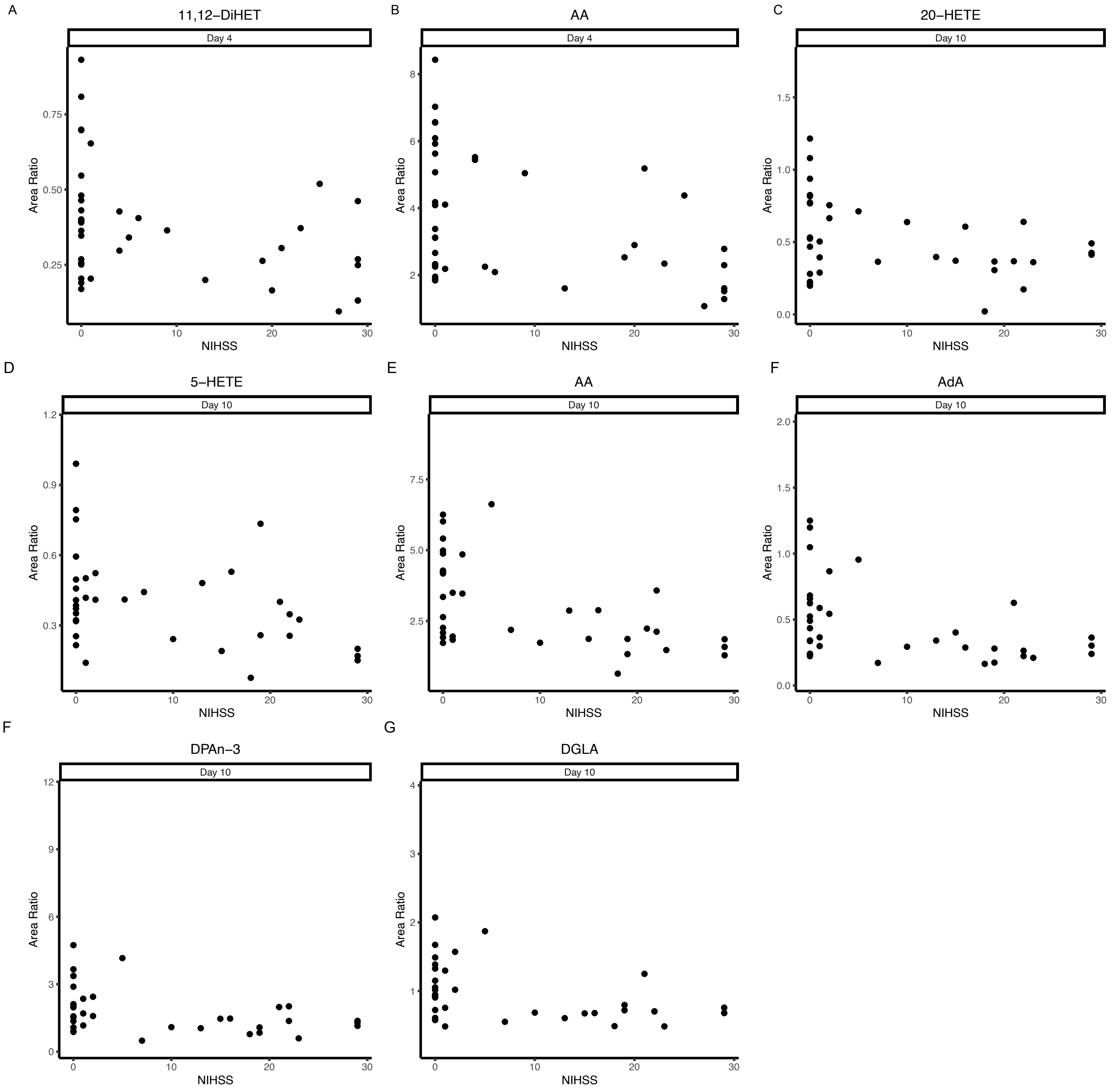

Supplementary Figure 8

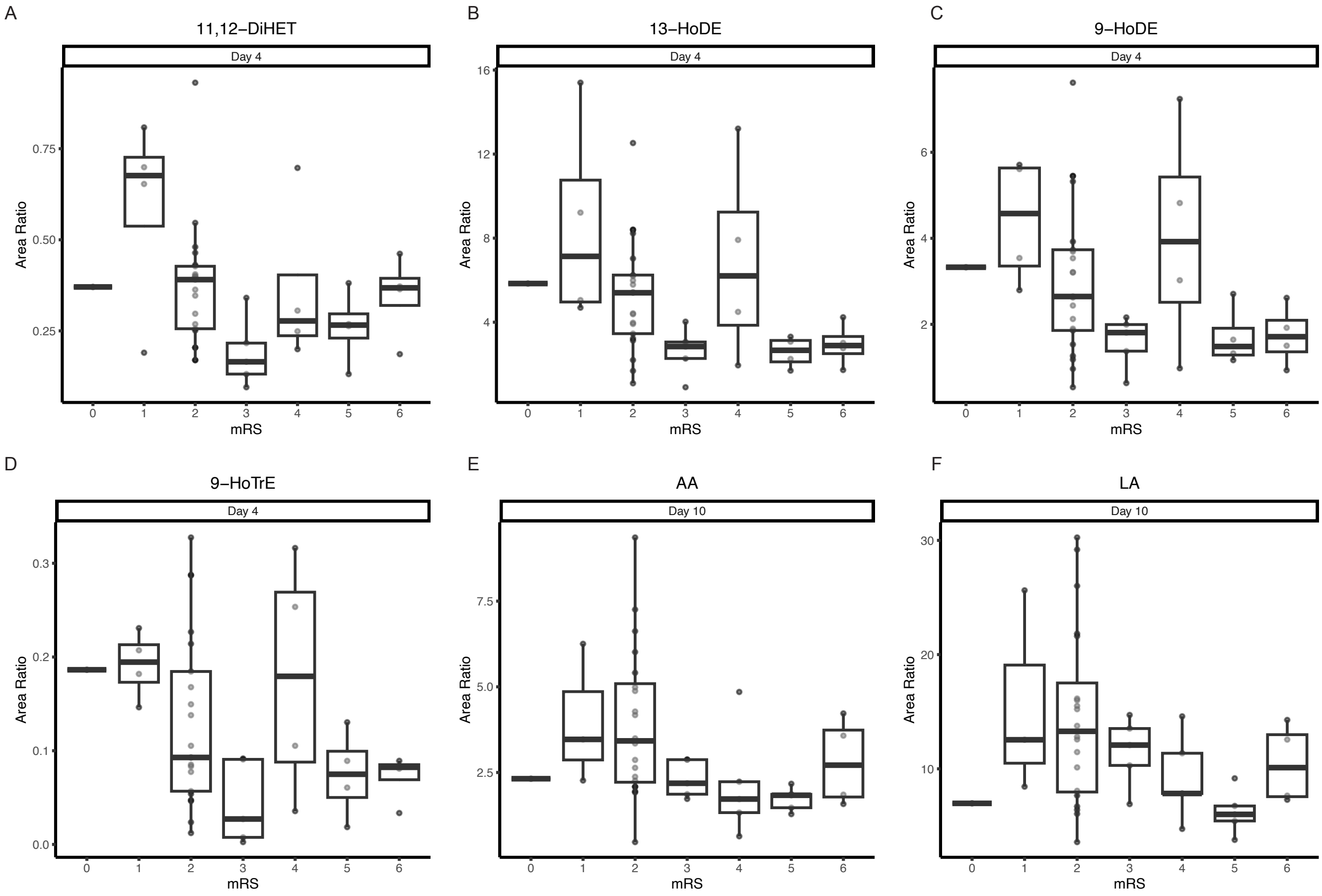

Supplementary Figure 9
